# Spiral spin liquid noise

**DOI:** 10.1073/pnas.2422498122

**Published:** 2025-03-18

**Authors:** Hiroto Takahashi, Chun-Chih Hsu, Fabian Jerzembeck, Jack Murphy, Jonathan Ward, Jack D. Enright, Jan Knapp, Pascal Puphal, Masahiko Isobe, Yosuke Matsumoto, Hidenori Takagi, J. C. Séamus Davis, Stephen J. Blundell

**Affiliations:** ^a^Department of Physics, Clarendon Laboratory, University of Oxford, Oxford OX1 3PU, United Kingdom; ^b^Max Planck Institute for Chemical Physics of Solids, Dresden D-01187, Germany; ^c^School of Physics, University College Cork, Cork T12 R5C, Ireland; ^d^Max Planck Institute for Solid State Research, Stuttgart D-70569, Germany; ^e^Department of Physics, Cornell University, Ithaca, NY 14853

**Keywords:** spin noise spectroscopy, spiral spin liquid, quantum spin liquid

## Abstract

An emerging concept for identification of different types of spin liquids [C. Broholm *et al.*, *Science* **367**, eaay0668 (2020)] is through the use of spontaneous spin noise [S. Chatterjee, J. F. Rodriguez-Nieva, E. Demler, *Phys. Rev. B* **99**, 104425 (2019)]. Here, we develop spin noise spectroscopy for spin liquid studies by considering Ca_10_Cr_7_O_28_, a material hypothesized to be either a quantum or a spiral spin liquid (SSL). By enhancing techniques introduced for magnetic monopole noise studies [R. Dusad *et al.*, *Nature* **571**, 234–239 (2019)], we measure the time and temperature dependence of spontaneous flux Φ(t,T) and thus magnetization M(t,T) of Ca_10_Cr_7_O_28_ samples. The resulting power spectral density of magnetization noise $S_{M}\left(\omega ,T\right)$ reveals intense spin fluctuations with $S_{M}\left(\omega ,T\right)\propto \omega^{-\alpha (T)}$ and $0.84<\alpha \left(T\right)<1.04$. Both the variance $\sigma_{M}^{2}\left(T\right)$ and the correlation function $C_{M}\left(t,T\right)$ of this spin noise undergo crossovers at a temperature $T^{*}\approx 450\;\mathrm{mK}$. While predictions for quantum spin liquids are inconsistent with this phenomenology, those from Monte–Carlo simulations of a two-dimensional (2D) SSL state in Ca_10_Cr_7_O_28_ yield overall quantitative correspondence with the measured frequency and temperature dependences of $S_{M}\left(\omega ,T\right),\;C_{M}\left(t,T\right)$, and $\sigma_{M}^{2}\left(T\right)$, thus indicating that Ca_10_Cr_7_O_28_ is an SSL.

## Significance

No state of matter can be defined by what it is not; yet spin liquids are often conjectured to exist based only on nonexistence of magnetic order. An emerging concept designed to circumvent this ambiguity is to identify each spin liquid type by its spontaneous spin noise. Here, we develop spin noise spectroscopy for studies of Ca_10_Cr_7_O_28_, a material hypothesized variously to be either a quantum or spiral spin liquid (SSL). At sub-Kelvin temperatures, we detect and quantify intense spin-noise spectra that, due to their general consistency with Monte–Carlo simulations, provide evidence that Ca_10_Cr_7_O_28_ is an SSL. The utility of spin noise spectroscopy for fingerprinting a nonordered spin state demonstrated here opens a general approach to spin liquid research.

In theory, spin liquids can occur in either classical (1) or quantum (2, 3) incarnations. The former exhibits massive ground-state degeneracy of its spin configurations, while the latter exhibits quantum entanglement of its localized spins along with fractionalized spin excitations. Candidate materials for either of these states are most often designated based on the absence of long-range magnetic order when the energy scale of magnetic interactions greatly exceeds that of temperature (1–3). But no spin liquid can be identified conclusively in this way, meaning that innovative techniques are urgently required to specify each material’s spin liquid state. A promising concept is to measure the unique spectrum of spontaneous spin noise generated by quantum and thermal fluctuations, thereby “fingerprinting” each spin liquid state (4–9). For example, in fermionic atomic vapors, the variance of magnetization-noise $\sigma_{M}^{2}\equiv \left\langle M^{2}(t)\right\rangle$ distinguishes the Bardeen–Cooper–Schrieffer superfluid state from the Bose–Einstein condensate state and from the antiferromagnetic state (4). Or, for the case of random exchange coupling Heisenberg spin-1/2 phases, theory predicts magnetization noise exhibiting $S_{M}\left(\omega \right)\propto \omega^{-\alpha }$ with 0.5<α<1 due to finite temperature many-body-localization (6). Finally, for a canonical *U*(1) gapless quantum spin liquid state with a spinon Fermi surface, theory predicts white magnetization noise for which $S_{M}\left(\omega \right)$ is a constant (8, 9).

The utility of this approach has recently been demonstrated (10–14) for the case of emergent magnetic monopoles (15–17) in spin ice, e.g. Dy_2_Ti_2_O_7_ and Ho_2_Ti_2_O_7_, and in artificial spin ices. There, thermally activated spin flips generate emergent magnetic monopole charges ±m. Generation-recombination theory for these monopoles then predicts magnetization noise $S_{M}\left(\omega ,T\right)=4\sigma_{M}^{2}\left(T\right)\tau \left(T\right)/\left(1+{\left(\omega \tau (T)\right)}^{2}\right)$ where ω and T are angular frequency and temperature, $\sigma_{M}^{2}\left(T\right)$ is the magnetization variance and $\tau \left(T\right)$ is relaxation time (18). Congruently, Monte Carlo (MC) simulations from the realistic spin-ice Hamiltonian (19) predict a closely related magnetization noise spectrum $S_{M}\left(\omega ,T\right)\propto \tau \left(T\right)/\left(1+{\left(\omega \tau (T)\right)}^{b(T)}\right)$ where $b\left(T\right)<2$ because of correlations in the monopole motion (10, 20). Most recently, discovery of the dynamical fractal nature of monopole trajectories (12) yielded the prediction that $S_{M}\left(\omega ,T\right)\propto \tau \left(T\right)/\left(1+{\left(\omega \tau (T)\right)}^{b(T)}\right)$, with $b\left(T\right)=1.5$ because there are two different possible microscopic spin-flip rates. By now, virtually all key predictions of spin noise theories (10, 12, 18) specific to the monopole dynamics in spin-ice pyrochlores have been borne out directly in SQUID-based spin noise spectroscopy experiments (10, 11, 13, 14) that measure $S_{M}\left(\omega ,T\right)$ and correlation function $C_{M}\left(t,T\right)$ of Dy_2_Ti_2_O_7_. Evidently, these achievements provide motivation to deploy this technique more generally for spin liquid research.

## Ca_10_Cr_7_O_28_ Spin Liquid

**Fig. 1.**
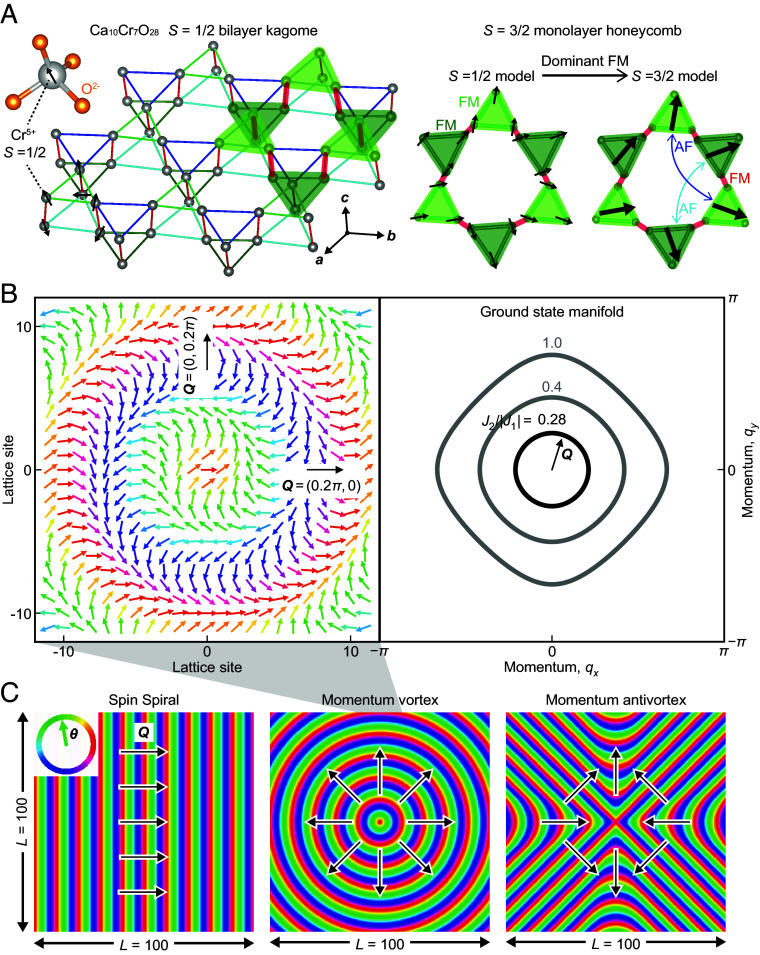
Ca_10_Cr_7_O_28_ SSL and momentum vortex. (*A*) *Left*: Schematic of the Ca_10_Cr_7_O_28_ distorted bilayer kagome lattice. Each Cr^5+^ ion hosts spin-1/2 under a tetragonal crystal field. The six Cr^5+^ spin-1/2 states per unit cell occur at the sites shown. *Right*: Three spins on a triangular plaquette on each layer are bound by a strong ferromagnetic interaction, and they form a frustrated spin-3/2 on a monolayer honeycomb network with ferromagnetic nearest-neighbor and antiferromagnetic next-nearest-neighbor interactions (26). (*B*) *Left*: Schematic of a spin spiral state in which the angle to the x-axis of the spin vector ϑ at point r is $\theta \left(r\right)$. In an SSL, the spin spiral ground-state wavevector Q indicated by a black arrow is free to point at any in-plane angle Θ. The arrangement shown here is of a unique topological defect referred to as momentum vortex, such that the line-integral on any trajectory surrounding the symmetry point is ∮∇Θ·dl=2π. *Right*: Contours of degenerate ground-state wavevector Q in the plane for different parameterizations of the Hamiltonian Eq. **1**. (*C*) Schematic images of θ(r) for three simple cases. *Left*: A topological defect-free spin spiral state. *Center*: A simple momentum vortex fixed at the origin. *Right*: A simple momentum antivortex.

To do so, we study Ca_10_Cr_7_O_28_ (21–25), a quasi-2D material consisting of weakly coupled bilayers (Fig. 1*A*). Each is a buckled kagome lattice in which the triangular plaquettes have alternating sizes (22). The magnetic Cr^5+^ ions have a six-site unit cell, with each Cr^5+^ located within a distorted CrO_4_ tetrahedron and having a singly occupied S=1/2 state (Fig. 1*A*). The isotropic magnetic susceptibility of Ca_10_Cr_7_O_28_ exhibits a Curie–Weiss temperature $T_{\mathrm{CW}}=+2.35\;K$ indicative of ferromagnetic interactions on the J≲1 meV energy scale (23). The zero-field magnetic specific heat capacity C(T) has a broad maximum at T≈3 K with a sharper peak followed by precipitous drop below $T^{*}\approx 450\;\mathrm{mK}$ (21). For $T<T^{*}$, it exhibits an approximately linear temperature dependence C(T)≅ηT (25). Zero-field muon spin rotation measurements exclude magnetic order down to T≈20 mK and instead evidence spin fluctuations which slow down on cooling and become persistent below T* (21). Inelastic neutron-scattering detects a spin excitation spectrum Σ(q,E) lacking well-defined spin-wave modes (25) but with a ring-like closed contour of scattering at intermediate energies (21, 23, 25).

## Spin Liquid Scenarios for Ca_10_Cr_7_O_28_

Two distinct spin liquid scenarios have been envisioned to explain the ${T<T}^{*}$ state. The first hypothesis is that Ca_10_Cr_7_O_28_ is a quantum spin liquid (QSL). No magnetic order whatsoever is detected at temperatures down to T≈20 mK, thermodynamically, by elastic/inelastic neutron scattering, or by muon spin precession. The relaxation rates of muon polarization $P\left(t\right)$ demonstrate that the spins remain entirely dynamic to the same low temperature (21). A continuum of dispersionless spin excitations exists along with a diffuse ring of intense scattering at ℏω∼0.3 meV, all confined to the kagome plane. Further, based on the linear temperature dependence of magnetic specific heat, a QSL with *Z*_2_ symmetry and a spinon Fermi surface has been inferred (25). Finally, pseudofermion functional renormalization group theory (21) or tensor network theory (27) both indicate a quantum magnetic ground state. The consequent principal hypothesis has been that Ca_10_Cr_7_O_28_ is a QSL (21–25, 27). On the other hand, it has been conjectured that Ca_10_Cr_7_O_28_ is a spiral spin liquid (SSL). In this state (26, 28–40) a spin spiral occurs at wavevector ***Q***, during each modulation of which the spin direction undergoes a spiral evolution (Fig. 1*B*). In an SSL, the vectorial direction ***Q*** is not fixed but occupies a continuous closed contour (28, 35, 37) in reciprocal space (Fig. 1*B*). Such systems with a subextensive degeneracy avoid long-range ordering (41) and are distinct from any static magnetically ordered state with well-defined spin wave modes. The SSL state has been mainly studied in a classical context and is typically conceived to be in a classical SSL state. Accordingly, we consider the classical situation throughout this paper, although we note that the interplay of quantum effects and the degenerate spiral manifold remains an open question as a nonordered “quantum SSL” is a theoretical possibility (42, 43). The Ca_10_Cr_7_O_28_ model of spin-1/2 on a distorted bilayer kagome can be mapped to interacting spin-3/2 on a monolayer honeycomb lattice (Fig. 1*A*), when 3 spins on alternative triangular plaquettes form a *S* = 3/2 state by ferromagnetic interactions (25, 26, 34) so that the expected spin behavior is closer to the classical limit. This model is frustrated, and its MC simulation predicts an SSL with q-space ring-like correlations (26, 34) consistent with the experiment (21, 23, 25). Generic 2D XY models for the SSL state further predict the existence of a unique topological defect referred to as a momentum vortex (37) which could dominate the low-energy physics. This defect occurs at a point around which the SSL continuously occupies all possible ***Q***-vector states on its manifold (Fig. 1*C*). MC simulations predict that, as a result of the nonlocality of these topologically constrained momentum vortices, dynamics slows as temperature is decreased and eventually reaches a metastable configuration. Consequently, the evolution is from a trivial paramagnet into a “pancake” liquid state and eventually into an SSL before freezing into a vortex lattice (37). Thus, on the basis of a nonordered dynamic spin state having diffuse spin correlations seen in a closed contour of more intense neutron scattering, Ca_10_Cr_7_O_28_ is hypothesized to be an SSL (26, 34, 37). Based on the extant phenomenology of Ca_10_Cr_7_O_28_, however, it has not been possible to distinguish conclusively between these QSL and SSL scenarios.

## Spin Noise Predictions for Different Spin Liquids

To address this challenge using techniques introduced here, the spin noise spectra of a QSL and an SSL state are required. For *Z*_2_ and *U*(1) QSL, ref. 8 predicts frequency-independent noise power spectral density $S\left(\omega \right)\propto \omega^{0}$ in the high-temperature limit ω≪T where our studies are carried out (*SI Appendix*). For SSL on the other hand rapid theoretical advances (37) have occurred recently using MC simulations based on a generic XY model for a 2D SSL with Hamiltonian

[1] $$\begin{array}{c} H=J_{1}\sum_{<ij\;{>}_{1}}\vartheta_{i}\cdot \vartheta_{j}+J_{2}\sum_{<ij\;{>}_{2}}\vartheta_{i}\cdot \vartheta_{j}+J_{3}\sum_{<ij\;{>}_{3}}\vartheta_{i}\cdot \vartheta_{j}. \end{array}$$

**Fig. 2.**
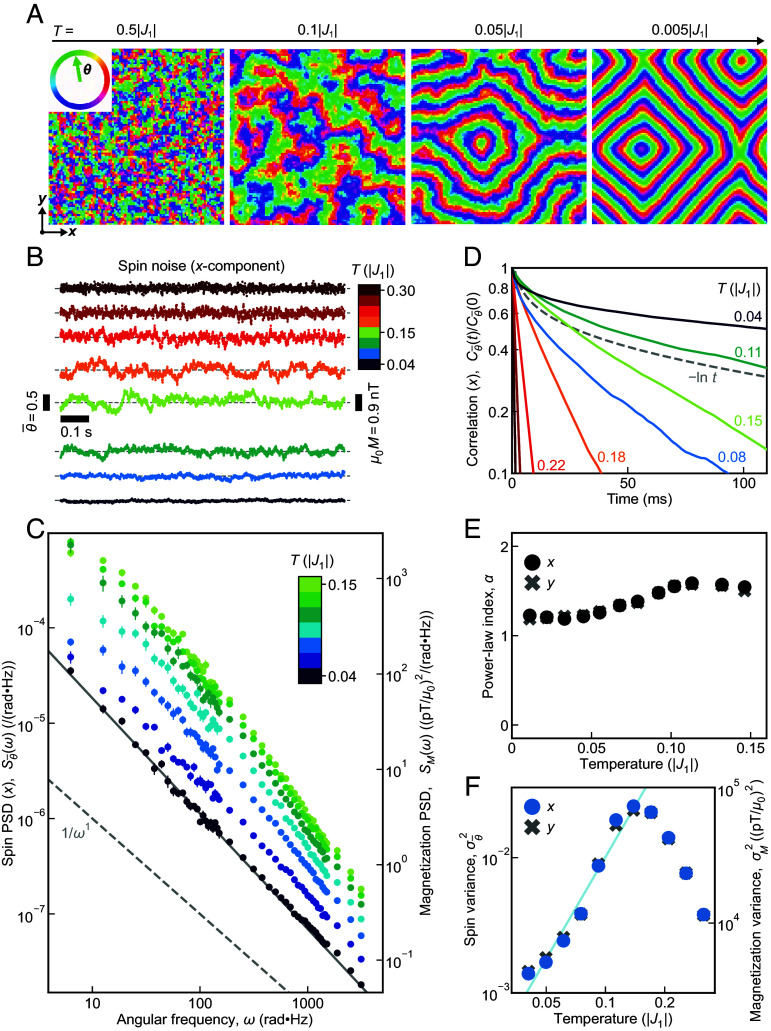
MC simulations of SSL noise for Ca_10_Cr_7_O_28_ relevant parameters. (*A*) MC simulations of a snapshot of θ(r) on the SSL model of Eq. **1** and ref. 37 using a square lattice with N=100×100 sites r, each site with an in-plane spin unit vector ϑ(r). Each snapshot is for a different temperature so that this sequence of SSL simulation snapshots is for approximately *T* = 0.5$\left|J_{1}\right|$, 0.1$\left|J_{1}\right|$, 0.05$\left|J_{1}\right|$ and 0.005$\left|J_{1}\right|$. While the spins point random directions in a paramagnet state at high temperature, they become spatially correlated at *T* = 0.1 $\left|J_{1}\right|$ corresponding to a pancake liquid state. At *T* = 0.05$\left|J_{1}\right|$, the system is in an SSL state with spiral domains and momentum vortices, which become clearer and more rigid upon further cooling (37). Examples of these results are demonstrated in Movies S1 and S2. (*B*) MC predicted time sequence of average x-component spin ${\bar{\vartheta }}_{x}\left(t,T\right)=\frac{1}{N}\sum_{r}\vartheta_{x}\left(r,t,T\right)$ at eight temperatures for N=40×40 sites. The average spin fluctuates at a second timescale and the amplitude of low-frequency noise grows as the system is cooled down to *T* = 0.15$\left|J_{1}\right|$. Below *T* = 0.15$\left|J_{1}\right|$ the noise amplitude gradually diminishes. We take 1 MC time step to be τ=1 μs. ${\bar{\vartheta }}_{x}\left(t,T\right)$ is down-sampled for visual clarity to every 500 MC steps so that time intervals shown here are 500τ=500 μs. The frequency component above 1 kHz is filtered out. The magnitude of magnetization noise estimated as described in *SI Appendix* is indicated by the bar on the right. The simulated ${\bar{\vartheta }}_{y}\left(t,T\right)$ results are statistically equivalent as shown in *SI Appendix*, Fig. S2*A*. (*C*) From the time sequences ${\bar{\vartheta }}_{x}\left(t,T\right)$ described in (*B*), the power spectral density of simulated SSL noise $S_{{\bar{\vartheta }}_{x}}\left(\omega ,T\right)$ is derived as a function of temperature T and shown for seven selected temperatures. Again we take 1 MC time step to be τ=1 μs. Here, the error bars are the SE of the independent MC simulation runs. The anticipated power spectral density of magnetization noise $S_{M}\left(\omega ,T\right)$ is shown on the right-hand axis as estimated from calculations described in *SI Appendix*. The spectrum shows a powerful low-frequency noise down to ω/2π=1 Hz with a diminishing power below $T=0.15\left|J_{1}\right|$. An example of fitted $\omega^{-\alpha }$ line (gray) is drawn and obtained α values are plotted in (*E*). (*D*) From the time sequences ${\bar{\vartheta }}_{x}\left(t,T\right)$ described in (*B*), the correlation function of simulated SSL noise $C_{{\bar{\vartheta }}_{x}}\left(t,T\right)/C_{{\bar{\vartheta }}_{x}}\left(0,T\right)$ is derived. The gray dashed line is an exemplary -lnt curve $\left(1-0.15\text{ln}(t(\text{ms}))\right)$. (*E*) By fitting the power spectral density of simulated SSL noise to $S_{{\bar{\vartheta }}_{x,y}}\left(\omega ,T\right)\propto \omega^{-\alpha (T)}$, the SSL noise power-law α(T) is derived and found to be $\alpha \left(T\right)\approx 1.2$ at the lowest temperature. (*F*) From the time sequences ${\bar{\vartheta }}_{x,y}\left(t,T\right)$ in (*B*), the variance of simulated SSL noise $\sigma_{{\bar{\vartheta }}_{x,y}}^{2}\left(T\right)$ is presented. The variance peaks around $T=0.15\left|J_{1}\right|$ and diminishes approximately as $T^{2.5}$ (blue line).

Here, $\vartheta_{i}$ represents XY spins constrained to lattice sites in a plane, and $J_{1};\;J_{2};\;J_{3}$ are the first, second, and third nearest neighbor spin couplings. For parameters $J_{1}=-1;J_{2}>1/4;J_{3}=J_{2}/2$ this system is an SSL exhibiting a ground-state set of spiral density waves with momenta ***Q*** satisfying $2\cos^{2}Q_{x}+2\cos^{2}Q_{y}+4cosQ_{x}cosQ_{y}=1/2J_{2}^{2}$: This is a continuous closed contour in *q*-space within the first reciprocal unit cell of the lattice (Fig. 1*B*). The full details of our MC simulations are presented in *SI Appendix* using Eq. **1** with $J_{1}=-1.0;$ $J_{2}=0.28;J_{3}=0.14$ for an array of N=L×L spins on a square lattice with periodic boundary conditions. Such semiclassical simulation results using *S* = 3/2 for Ca_10_Cr_7_O_28_ are well supported by successful previous studies (26, 34) (*SI Appendix*). Equilibration of ϑ(r,T), the in-plane spin vector at each site ***r***, to temperature T uses an initial set of ϑ(r) randomly selected with uniform probability. Examples of ϑ(r,T) evolution in the equilibration process can be visualized in Movies S1 and S2. At each temperature, this yields a representative spin configuration, four typical examples of which are shown for different T in Fig. 2*A*. The structure factor Σ(q) of each spin configuration is shown in *SI Appendix*, Fig. S11. This is in excellent agreement with the evolution from a paramagnet into a pancake liquid state, thence to an SSL and finally into a vortex lattice as reported by ref. 37.

Such thermalized configurations (Fig. 2*A*) subsequently initiate the simulation of $\vartheta \left(r,t,T\right)$ via the Metropolis Monte Carlo algorithm, which approximates the evolution of spins interacting with a thermal bath, i.e., thermal fluctuations of spins. Here, the system is evolved sequentially through $10^{7}$ MC time steps at each *T*. During MC simulations at $T∼0.15\left|J_{1}\right|$ we find the elementary local spin relaxation process occurs at a timescale ∼10 MC step (*SI Appendix*, Fig. S3). High-frequency AC susceptibility experiments yield a semicircular Cole–Cole plot indicating a microscopic spin relaxation time corresponding to ∼10 μs (21). Hence, setting each MC step to τ=1 μs simulates a microscopic relaxation time consistent with empirical observations; we then use a total MC simulation run time Γ=10 s. From these data, we predict the spin noise fingerprint of the SSL by calculating the x, y- components of the average spin ${\bar{\vartheta }}_{x,y}\left(t,T\right)=\frac{1}{N}\sum_{r}\vartheta_{x,y}\left(r,t,T\right)$ versus time, with typical examples shown in Fig. 2*B* and *SI Appendix*, Fig. S2*A*. Most importantly, the power spectral density of SSL spin noise is calculated from $\begin{array}{c} S_{{\bar{\vartheta }}_{x,y}}\left(\omega_{j},T\right)=\frac{1}{\pi \Gamma }{\left|\Delta t\sum_{k=0}^{K-1}e^{-i\omega_{j}t_{k}}{\bar{\vartheta }}_{x,y}\left(t_{k},T\right)\right|}^{2} \end{array}$, where Δt is time interval and K=Γ/Δt, with typical results shown in Fig. 2*C* and *SI Appendix*, Fig. S2*B*. With falling temperatures below $T=0.15\left|J_{1}\right|$, it shows strong low-frequency noise down to at least ω/2π=1 Hz that diminishes continuously in power. The correlation function of this SSL noise is $C_{{\bar{\vartheta }}_{x,y}}\left(t_{k},T\right)=\frac{1}{l_{ave}}\sum_{l=0}^{l_{ave}-1}{\bar{\vartheta }}_{x,y}$$\left(t_{l},T\right){\bar{\vartheta }}_{x,y}\left(t_{l}+t_{k},T\right)$, where $t_{k}=k\Delta t$ and $l_{ave}=9\times 10^{5}$; its typical temperature dependence is shown in Fig. 2*D* and *SI Appendix*, Fig. S2*C*. Well above $T=0.15\left|J_{1}\right|$, the correlations drop rapidly over time. As temperature falls, the decay of correlation slows down and the functional form becomes nearly $C_{{\bar{\vartheta }}_{x,y}}\left(t,T\right)\propto -\ln t$ (*SI Appendix*, Fig. S10*A*). To predict the temperature dependence of SSL noise power-law α(T), the $S_{{\bar{\vartheta }}_{x,y}}\left(\omega ,T\right)$ is fitted by a function $A\left(T\right)\omega^{-\alpha (T)}$ in the frequency range 1 Hz≤ω/2π≤500 Hz below $T=0.15\left|J_{1}\right|$ (*SI Appendix*, Figs. S1*B* and S2*D*), with the result shown in Fig. 2*E* that α(T)≈1.2±0.1 at the lowest temperature. Finally, the power-law β of the SSL noise variance  $\sigma_{{\bar{\vartheta }}_{x,y}}^{2}\left(T\right)\propto T^{\beta }$ below $T=0.15\left|J_{1}\right|$ is predicted in Fig. 2*F* using $\sigma_{{\bar{\vartheta }}_{x,y}}^{2}\left(T\right)=\frac{1}{K}$$\sum_{k=0}^{K-1}{\bar{\vartheta }}_{x,y}^{2}\left(t_{k},T\right)-{\left(\frac{1}{K}\sum_{k=0}^{K-1}{\bar{\vartheta }}_{x,y}\left(t_{k},T\right)\right)}^{2}$. The noise variance rapidly grows down to $T=0.15\left|J_{1}\right|$, then declines approximately as $T^{2.5}$. Simulation for Heisenberg spins using the same SSL Hamiltonian (*SI Appendix*) predicts quantitatively distinct spin noise features and the momentum vortex is harder to identify. Here, we mainly discuss the predictions for XY spins which, with the parameter set of our present simulations, conform best to the subsequently shown experimental observations in Ca_10_Cr_7_O_28_. Optimization of simulation parameters in a Heisenberg-spin simulation, which might alter its correspondence with the experiment, is left for future work. To summarize, our MC simulation for XY spins using Eq. **1** predicts that, for $T\leq 0.15\left|J_{1}\right|$, the noise spectrum of a generic 2D SSL has powerful spin fluctuations at least from 1 Hz to 500 Hz, a scale-invariant power spectral density $S_{{\bar{\vartheta }}_{x,y}}\left(\omega ,T\right)\propto \omega^{-1.2\pm 0.1}$, correlation functions $C_{{\bar{\vartheta }}_{x,y}}\left(t,T\right)\propto -\ln t$, and a noise variance $\sigma_{{\bar{\vartheta }}_{x,y}}^{2}\left(T\right)$ diminishing approximately as $T^{2.5}$.

## Spin Noise Spectroscopy of Ca_10_Cr_7_O_28_

**Fig. 3.**
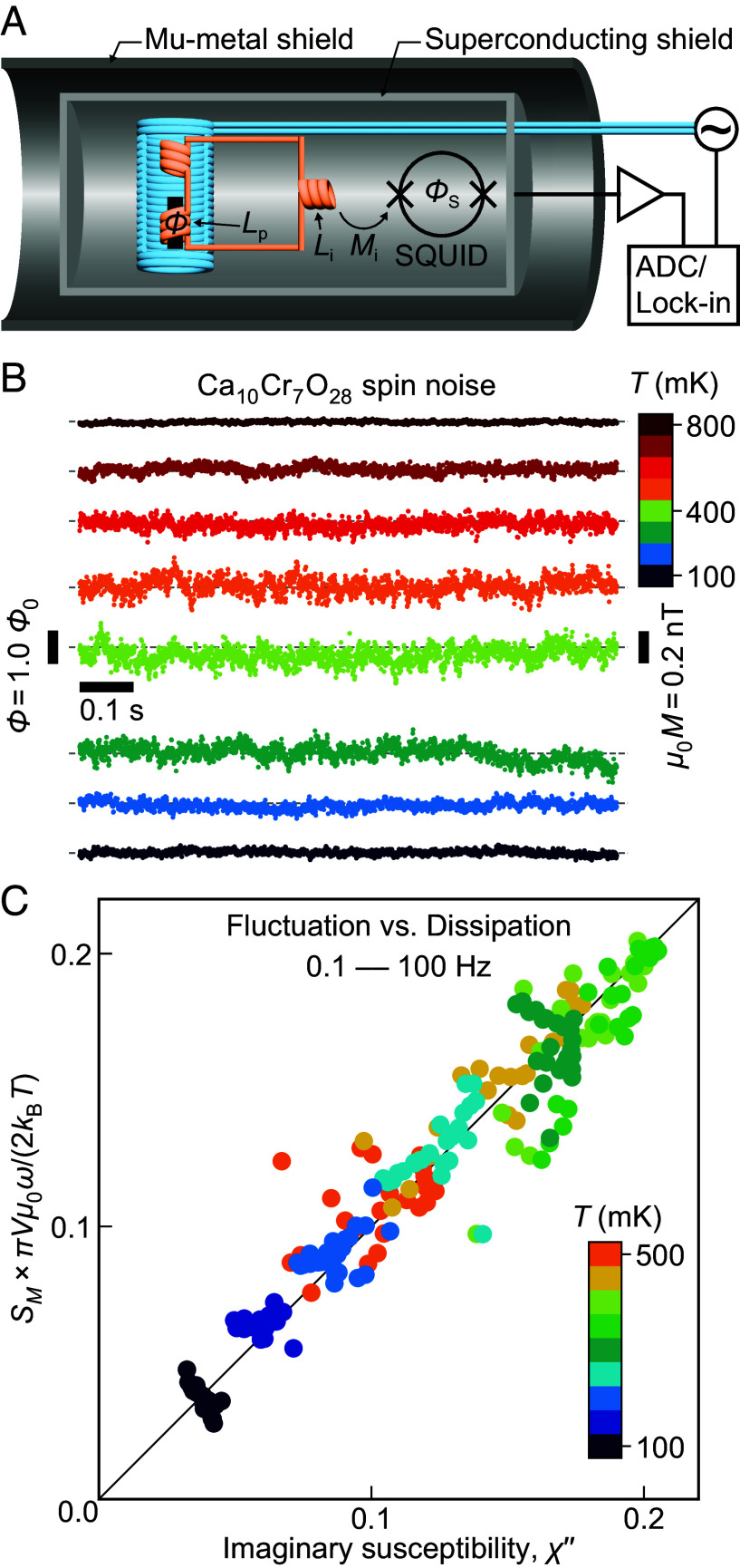
Spin noise measurements in Ca_10_Cr_7_O_28_. (*A*) Conceptual design of our spin noise spectrometer based on high-precision and high-bandwidth SQUID sensing of time-dependent flux Φ(t) generated by the Ca_10_Cr_7_O_28_ sample in the compensated superconductive pickup coil connected persistently to the SQUID input coil. (*B*) Eight typical time sequences of the measured magnetic flux Φ(t) generated by Ca_10_Cr_7_O_28_ at 100 mK≤T≤800 mK. The plotted datapoints are down-sampled to every 500 μs for visual clarity. The frequency above 1 kHz is filtered out. The equivalent spontaneous magnetization noise at the sample $\mu_{0}M\left(t\right)$ is very intense, reaching almost the nT scale. (*C*) Simultaneously measured magnetization noise power spectral density $S_{M}\left(\omega ,T\right)$ and imaginary part of AC susceptibility $\chi^{\prime \prime }(\omega ,T)$of Ca_10_Cr_7_O_28_ plotted as $\pi V\mu_{0}\omega S_{M}(\omega ,T)/2k_{B}T$ versus $\chi^{\prime \prime }(\omega ,T)$ over the frequency range 0.1 Hz≤ω/2π≤100 Hz and temperature range 100 mK ≤ *T* ≤ 500 mK. This indicates that the fluctuation-dissipation theorem $\chi^{\prime \prime }(\omega ,T)=\pi V\mu_{0}\omega S_{M}(\omega ,T)/2k_{B}T$ is predominantly valid, and that the spin liquid state in Ca_10_Cr_7_O_28_ remains in dynamical equilibrium down to at least 0.1 Hz.

To explore these predictions, we perform SQUID-based flux-noise spectrometry (10, 11) achieving magnetic field sensitivity approaching $\mu_{0}\delta M{\leq 10}^{-14}\;T/\sqrt{Hz}$ (Fig. 3*A*), and using cryogen-free ultralow-vibration refrigerators in the range 10 mK ≤ *T* ≤ 5,000 mK (*SI Appendix*). The time sequence of the magnetic flux $\Phi \left(t\right)$ generated by the sample magnetization $M\left(t\right)=c\Phi (t)$ within the pickup coil is measured with microsecond precision via a persistent superconducting circuit that transforms it into the flux $\Phi_{S}\left(t\right)$ at the SQUID input coil as

[2] $$\begin{array}{c} \Phi_{S}\left(t\right)=(M_{i}/(L_{p}+L_{i}))\Phi \left(t\right)=\\ (M_{i}/(L_{p}+L_{i}))c^{-1}M\left(t\right)\equiv c_{S}^{-1}M\left(t\right). \end{array}$$

Here, $L_{p}$ is a pickup coil inductance, $L_{i}$ is a SQUID-input coil inductance, $M_{i}$ is a mutual inductance between the SQUID and its input coil, and $c_{S}$ is a constant set by the geometry of each pickup coil (Fig. 3*A*). Hence, the output voltage of the SQUID $V_{S}\left(t\right)$ is related to magnetization $M\left(t\right)$ as

[3] $$V_{S}\left(t\right)=g\Phi_{S}\left(t\right)=gc_{S}^{-1}M\left(t\right)\equiv a^{-1}M\left(t\right),$$

where g is the total gain of the electronics. For a given experiment, the value of a can be calibrated accurately (*SI Appendix*). The time sequences of magnetization fluctuations are recorded from $V_{S}\left(t\right)$ at each temperature *T* as $M\left(t,T\right)=aV_{S}\left(t,T\right)$ from whence the power spectral density of magnetization noise $S_{M}\left(\omega ,T\right)\equiv a^{2}S_{V_{S}}\left(\omega ,T\right)$ can be derived.

Our Ca_10_Cr_7_O_28_ samples are prepared by the traveling-solvent-floating-zone method (22). The lattice structure is confirmed by X-ray Laue diffraction, and Curie–Weiss fit $\chi =\chi_{0}+\frac{C_{Curie}}{T-T_{CW}}$ of the DC magnetic susceptibility in the temperature range 50 to 250 K yields the Curie–Weiss temperature $T_{\mathrm{CW}}=+2.6\;K$ and an effective magnetic moment $\mu_{\text{eff}}\approx \;1.69\;\mu_{B}$ (*SI Appendix*, Fig. S5*B*) (23). Typical examples of the mm-scale Ca_10_Cr_7_O_28_ single crystals studied are shown in *SI Appendix*, Fig. S5*A*. The experimental setup is integrated into either a cryogen-free ^3^He refrigerator or cryogen-free ^3^He/^4^He dilution refrigerator spanning the temperature range 10 mK ≤ *T* ≤ 5,000 mK. Immediately upon commencing these experiments, we found that Ca_10_Cr_7_O_28_ generates powerful magnetization noise. Fig. 3*B* shows exemplary time sequences of the measured magnetic flux Φ(t,T) generated by Ca_10_Cr_7_O_28_ for eight selected temperatures demonstrating the intense magnetization amplitude fluctuations approaching nT amplitudes. These data are digitized by an effective 16-bit analog-to-digital converter with acquisition time interval of minimum $\Delta t=t_{k+1}-t_{k}=1\;\mu s$, yielding a sequence of values $\Phi (t_{k},T)$ over a continuous time epoch Γ. From this, we derive the power spectral density

**Fig. 4.**
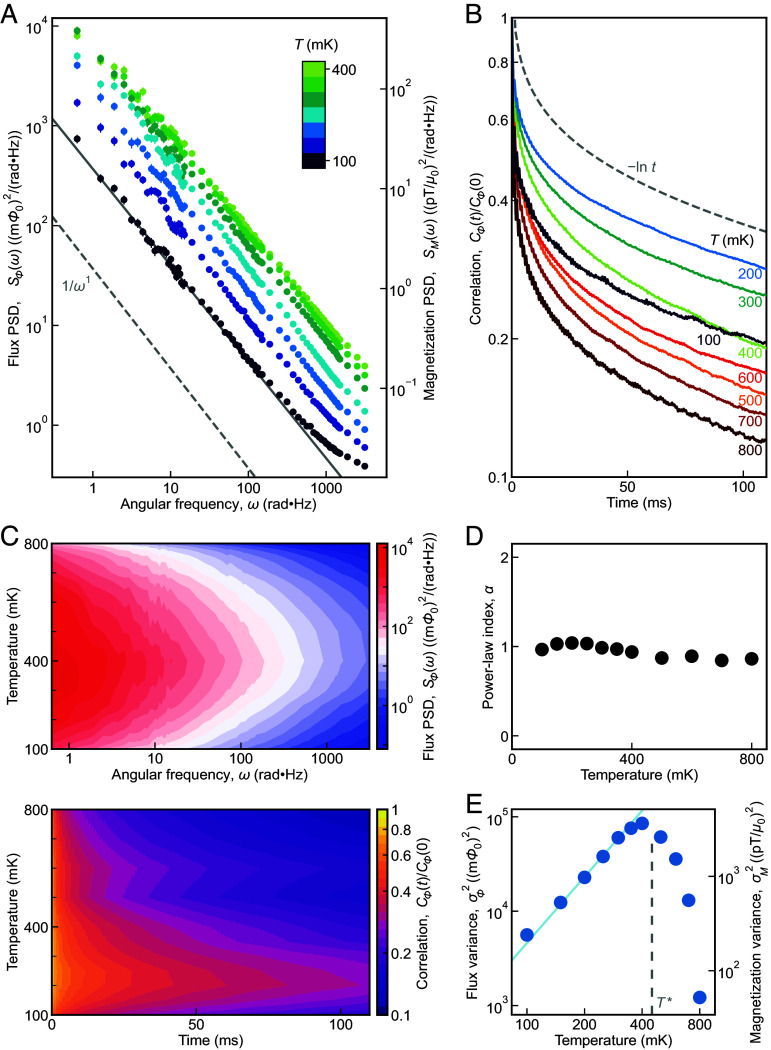
SSL noise of Ca_10_Cr_7_O_28_. (*A*) Typical measured power spectral density of flux noise $S_{\Phi }(\omega ,T)$ generated by Ca_10_Cr_7_O_28_ samples for the temperature range 100 mK≤T≤400 mK. Here, the error bars are the SE of separated segments (*SI Appendix*). The noise spans a broad frequency range of at least 0.1 Hz≤ω/2π≤500 Hz. The equivalent power spectral density of magnetization noise at the sample $S_{M}\left(\omega ,T\right)$ is presented in units of Tesla on the right-hand axis. An example of fitted $\omega^{-\alpha }$ line (gray) is shown and obtained α is plotted in (*D*). These data may be compared to the expected SSL spin noise spectra predicted in Fig. 2*C*. (*B*) Measured normalized correlation function $C_{\Phi }(t,T)/C_{\Phi }(0,T)$ of flux noise Φ(t) generated by Ca_10_Cr_7_O_28_ for the temperature range 100 mK≤T≤800 mK. The gray dashed line is an exemplary -lnt curve $\left(1-0.14\text{ln}(t\left(\text{ms}\right))\right)$. This may be compared with the expected temperature dependence of SSL correlation functions predicted in Fig. 2*D*. (*C*) *Top*: Overall power spectral density of flux noise $S_{\Phi }(\omega ,T)$ generated by Ca_10_Cr_7_O_28_ samples for the temperature range 100 mK≤T≤800 mK. The noise power is the strongest at ω/2π=0.1 Hz around 400 mK and gradually declines as the temperature gets away and frequency gets higher. *Bottom*: Evolution of $C_{\Phi }(t,T)/C_{\Phi }(0,T)$ for the magnetic flux noise Φ(t) generated by Ca_10_Cr_7_O_28_ at 100 mK≤T≤800 mK. (*D*) Measured noise power-law index $\alpha \left(T\right)$ obtained by fitting power spectral density $S_{\Phi }\left(\omega ,T\right)=A\left(T\right)\omega^{-\alpha \left(T\right)}$ in the range 0.1 Hz≤ω/2π≤20 Hz. This is to be compared with the expected temperature dependence of SSL noise power-law index predicted in Fig. 2*E*. (*E*) Temperature dependence of measured flux noise variance $\sigma_{\Phi }^{2}\left(T\right)$ calculated from time series in Fig. 3*B* showing a crossover peak around $T^{*}\approx 450\;\text{mK}$. The equivalent magnetization noise variance $\sigma_{M}^{2}\left(T\right)$ is on the right axis. The noise diminishes for T≤300 mK with an approximate power law $T^{2.3}$ (blue line). These data can be compared to the expected temperature dependence of SSL noise variance $\sigma_{\bar{\vartheta }}^{2}\left(T\right)$ as predicted in Fig. 2*F*.

[4] $$\begin{array}{c} S_{\Phi }\left(\omega_{j},T\right)\equiv \frac{1}{\pi \Gamma }{\left|\Delta t\sum_{k=0}^{K-1}e^{-i\omega_{j}t_{k}}\Phi \left(t_{k},T\right)\right|}^{2}, \end{array}$$

and consequently $S_{M}\left(\omega ,T\right)=c^{2}S_{\Phi }\left(\omega ,T\right)$. Next, we carry out a sequence of measurements consisting of varying the sample temperature from 100 mK to 800 mK in steps of 50 or 100 mK and measuring $\Phi (t_{k},T)$ with Γ=1,000 s at each temperature. From that dataset, the $S_{\Phi }\left(\omega ,T\right)$ for the temperature range 100 mK ≤ *T* ≤ 800 mK are derived using Eq. **4**. These $S_{\Phi }(\omega ,T)$ spectra at $T\leq T^{*}$ are shown in Fig. 4*A* in the frequency range 0.1 Hz ≤ω/2π≤ 500 Hz. The full temperature range and full frequency up to 50 kHz are shown in *SI Appendix*, Figs. S7*A* and S8. The equivalent power spectral density of magnetization noise $S_{M}\left(\omega ,T\right)$ in units of Tesla $\left({M=B/\mu }_{0}\right)$ is shown at right. Even at this elementary stage the phenomenology of Ca_10_Cr_7_O_28_ appears quite remarkable because powerful fluctuations in the spin-1/2 magnetization of a mm-scale sample are spontaneously generating magnetic fields approaching $10^{-10}$ T and occur in a frequency range 0.1 Hz≤ω/2π≤50 kHz. Most profoundly, the $S_{M}\left(\omega ,T\right)$ is obviously scale invariant $S_{M}\left(\omega ,T\right)\propto \omega^{-\alpha (T)}$, and the noise power diminishes precipitously below $T^{*}$ (Fig. 4*A*).

We also measure the Ca_10_Cr_7_O_28_ magnetic susceptibility $\chi \left(\omega ,T\right)\equiv \frac{\mu_{0}M\left(\omega ,T\right)}{B\left(\omega \right)}=\chi^{\prime }\left(\omega ,T\right)+i\chi^{\prime \prime }\left(\omega ,T\right)$ simultaneously with $S_{M}\left(\omega ,T\right)$ (*SI Appendix*). The primary superconductive coil applies homogeneous axial AC magnetic fields $B\left(\omega \right)$, whose flux does not reach the SQUID due to the balanced astatic pair of coils in the flux pickup system (Fig. 3*A*). If the fluctuation–dissipation theorem holds for the spin liquid (as it would not for a spin glass) then $\chi^{\prime \prime }(\omega ,T)$ should equal $\pi V\mu_{0}\omega S_{M}(\omega ,T)/2k_{B}T$ where V is the sample volume (44). The simultaneously measured values of ${\pi V\mu }_{0}\omega S_{M}\left(\omega ,T\right)/2k_{B}T$, plotted versus $\chi^{\prime \prime }(\omega ,T)$ over the range 100 mK≤T≤500 mK, are presented in Fig. 3*C*. Evidently, the fluctuation–dissipation theorem holds and dynamical equilibrium is maintained in the spin liquid state of Ca_10_Cr_7_O_28_ at temperatures $T\ll T^{*}$, down to at least 0.1 Hz. Another characterization technique for magnetization noise is the correlation function $C_{\Phi }(t,T)$ which is evaluated directly from

[5] $$C_{\Phi }\left(t_{k},T\right)=\frac{1}{l_{\text{ave}}}\sum_{l=0}^{l_{\text{ave}}-1}\Phi \left(t_{l},T\right)\Phi \left(t_{l}+t_{k},T\right).$$

The normalized correlation function $C_{\Phi }(t,T)/C_{\Phi }(t=0,T)$ is shown in Fig. 4*B*. As temperature is lowered, the enhanced spin correlation grows becoming $C_{\Phi }(t,T)/C_{\Phi }(t=0,T)∼-\text{ln}t$ below T* (*SI Appendix*, Fig. S10*B*). Such a logarithmic decay of correlation function is quite distinct from that of any system with a single relaxation time where $C_{\Phi }\left(t,T\right)/C_{\Phi }\left(t=0,T\right)=\text{exp}(-t/\tau )$, and can imply a distribution of microscopic relaxation times with probabilities (45) $p\left(\tau \right)\propto 1/\tau$.

Fig. 4*A* presents the measured power spectral density of flux noise $S_{\Phi }(\omega ,T)$ generated by Ca_10_Cr_7_O_28_ samples for the temperature range 100 mK≤T≤400 mK. These $S_{\Phi }(\omega ,T)$ data may be compared to Fig. 2*C*. The measured correlation function $C_{\Phi }(t,T)/C_{\Phi }(0,T)$ of flux noise generated by Ca_10_Cr_7_O_28_ for 100 mK ≤ *T* ≤ 800 mK in Fig. 4*B* may be compared with Fig. 2*D*. In Fig. 4*C*, the upper panel shows contour plots of overall power spectral density of flux noise $S_{\Phi }(\omega ,T)$ generated by Ca_10_Cr_7_O_28_ samples for the temperature range 100 mK ≤ *T* ≤ 800 mK while the lower presents $C_{\Phi }(t,T)/C_{\Phi }(0,T)$ for the same range. The magnitude of power spectral density grows slowly down to $T^{*}$ and then diminishes rapidly below that. The coincidence of the crossover temperature $T^{*}$ indicates that the observed spin noise has the same origin as that of susceptibility (21), specific heat (21, 25), and muon spin rotation phenomenology (21). Fig. 4*D* shows the measured noise power-law index $\alpha \left(T\right)$ obtained by fitting data in Fig. 4*A* to the function $S_{\Phi }\left(\omega ,T\right)=A\left(T\right)\omega^{-\alpha \left(T\right)}$ in the range 0.1 Hz≤ω/2π≤20 Hz (*SI Appendix*, Fig. S7*B*) and this is to be compared with Fig. 2*E*. Finally in Fig. 4*E* we show the measured temperature dependence of flux noise variance $\sigma_{\Phi }^{2}\left(T\right)$ derived from Fig. 3*B*, showing a crossover peak at $T^{*}$ and diminution with power-law index β≈2.3±0.1; these data can be compared to the predicted temperature dependence of SSL noise variance $\sigma_{\bar{\vartheta }}^{2}\left(T\right)$ in Fig. 2*F*. The nonmonotonous rapid temperature dependence of spin noise indicates that its origin is thermal fluctuations, which is in line with the Monte Carlo simulation.

## Evidence for SSL in Ca_10_Cr_7_O_28_

There is impressive wide-ranging agreement between Monte–Carlo simulation predictions of SSL noise phenomena in Fig. 2 and the data in Fig. 4. First, magnetic field fluctuations near $10^{-10}$ T occur in a broad frequency range at least from 1 Hz≤ω/2π≤50 kHz. Second, the spin noise correlation function decays with a distinct form $C\left(t\right)∼-\ln t$. Third, the frequency power-law index of the spectral density $\alpha \left(T\right)$ reaches a value close to 1 at low temperatures. Finally, the magnetization noise variance grows upon cooling but then diminishes below the crossover T* with power-law index β≈2.5. On the other hand, the observed power spectral density characteristics are highly distinct from the $S\left(\omega \right)\propto \omega^{0}$ ($\alpha \left(T\right)=0$) as predicted for *Z*_2_ and *U*(1) QSLs (8, 9). Moreover, the combined phenomenology of temperature and frequency dependence observed in Ca_10_Cr_7_O_28_ spin noise is exceptional and appears inconsistent with the spin noise (5, 6, 8) due to quenched disorder (e.g., used to explain surface spins) or by a spin glass. Thus, the quantitative correspondence between the SSL simulations and the spin noise data, including for $S_{M}\left(\omega ,T\right),C_{M}\left(t,T\right)$, and $\sigma_{M}^{2}\left(T\right)$ over orders of magnitude in frequency, evidences the state of Ca_10_Cr_7_O_28_ as an SSL. Future studies exploring this conclusion will require extending the range of spin noise measurements to a higher frequency and performing comprehensive theoretical studies so that spin noise can be connected to the reported characteristics of neutron/muon studies. More broadly, the spin noise spectroscopy technique introduced to spin liquid studies here can indeed fingerprint a spin system, opening a promising avenue for spin liquid research.

## Supplementary Material

Appendix 01 (PDF)

Movie S1. **Visualization of the spin equilibration process in an *L* = 100 system**. The evolution of spin configuration as the *L* = 100 system is equilibrated by a total of 6 × 10^6^ MC steps. The color of the pixel represents the direction of the spin with the same color code as Fig. 2*A*. 10. MC steps are performed between each picture frame.

Movie S2. **Visualization of the spin equilibration process in an *L* = 40 system**. The evolution of spin configuration as the *L* = 40 system is equilibrated by a total of 6 × 10^6^ MC steps. The color of the pixel represents the direction of the spin with the same color code as Fig. 2*A*. 10^4^ MC steps are performed between each picture frame.

## Data Availability

Data (.csv) presented in this paper are deposited in Zenodo (46).
