# Supplementary material for: Spiral spin liquid noise
Source: Proc Natl Acad Sci U S A. 2025 Mar 18;122(12):e2422498122. doi: 10.1073/pnas.2422498122 (PMC11962441; doi:10.1073/pnas.2422498122)
Supplement: Supplementary file 1 — Appendix 01 (PDF) [file pnas.2422498122.sapp.pdf]

PNAS

Supporting Information for
Spiral spin liquid noise

Hiroto Takahashi, Chun-Chih Hsu, Fabian Jerzembeck, Jack Murphy, Jonathan Ward, Jack D. Enright, Jan Knapp, Pascal Puphal, Masahiko Isobe, Yosuke Matsumoto, Hidenori Takagi, J.C. Séamus Davis, Stephen J. Blundell

Email: jcseamusdavis@gmail.com & Stephen.Blundell@physics.ox.ac.uk

This PDF file includes:

Supporting text
Figures S1 to S11
Legends for Movies S1 to S2
SI References

Other supporting materials for this manuscript include the following:

Movies S1 to S2

Quantum spin liquid noise theory

'Fingerprinting' quantum spin liquids may, in theory, be achieved using their unique spectrum of spontaneous spin noise. For example, ref. 1 predicts spin noise spectra of various quantum spin liquids in different parameter regimes. A separate prediction is made for $\omega \ll T$ and $\omega \gg T$, and the $\omega \ll T$ regime is, at present, more relevant for our SQUID-based spin noise spectrometry. Another parameter controlling the noise spectrum is $d\omega/v$, where d and v are the measured length scale and spinon velocity, respectively. In the $\omega \ll T$ and $d\omega/v \ll 1$ regime more relevant for our experiment, the power spectral density $S(\omega, T)$ is predicted to be frequency-independent $S(\omega, T) \propto \omega^0$ for both Z_2 Dirac, Z_2 Fermi surface, and $U(1)$ Fermi surface quantum spin liquids, with different temperature dependences (1, 2).

Simulating time evolution of spins $\boldsymbol{\theta_i(t)}$ in a spiral spin liquid model

A classical Monte Carlo (MC) simulation, based on a generic XY model for a 2D spiral spin liquid (Eq. 1) with $J_1 = -1; J_2 = 0.28; J_3 = 0.14$, was performed for $N = L \times L$ spins on a square lattice with a periodic boundary condition. The equilibrated spin configuration at each temperature was prepared by annealing from high temperature. The initial direction of the spins was randomly selected with a uniform probability. The system was cooled down from $T = 2|J_1|$ to $0.005|J_1|$ via a step-by-step equilibration at selected temperatures $T = 2 \times 0.95^r |J_1|$ where $0 \le r \le 117$ (exponential cooling protocol).

Following ref. 3, two types of Monte Carlo updates were used to equilibrate the system. The first update is the standard Metropolis algorithm. A randomly selected spin attempts to flip to a new direction that is chosen with a uniform probability, and the flip is accepted with a probability of $\min(1, e^{-\Delta E/T})$ where ΔE is the change of total energy caused by the flip. The second used update is the over-relaxation update. A randomly selected spin $\boldsymbol{\vartheta}_i$ is reflected about a local exchange field $\boldsymbol{H}_i = \sum_j J_{ij}\, \boldsymbol{\vartheta}_j$, which is an energy conservation process that is empirically known to accelerate the equilibration process (4). One-third of the Monte Carlo updates are carried out by the Metropolis update, each of which is followed by two over-relaxation updates.

One MC step consists of N MC updates. 5×10^4 MC steps are performed at each temperature, amounting to a total of 6×10^6 MC steps. We performed equilibration for two system sizes L = 100 and L = 40. In Movie S1 (L = 100) and Movie S2 (L = 40), the equilibration process from $T = 2|J_1|$ to $0.005|J_1|$ is visualized, and representative spin configuration at four temperatures are shown in Fig. 2A. The corresponding structure factor $\Sigma(\boldsymbol{q}) = \frac{1}{N}\sum_{\boldsymbol{r}_i, \boldsymbol{r}_j} e^{i\boldsymbol{q}\cdot(r_i - r_j)} \langle \boldsymbol{\theta}_i \cdot \boldsymbol{\theta}_j \rangle$ is shown in Fig. S11. The final spin configuration at each temperature is recorded as an equilibrated state.

Starting from the obtained equilibrated spin configuration at each temperature, we simulated the time evolution of the spins. 10^7 MC steps consisting only of Metropolis updates are performed for the L = 40 system at each temperature. We set one MC step to $\tau = 1$ μs as

discussed in the section 'Comparing the simulation to a realistic system', making the total time of simulation $\Gamma = 10$ s.

The time evolution of average spin x- and y-components $\bar{\vartheta}_x(t_k, T) = \frac{1}{N}\sum_i \vartheta_i^x\,(t_k, T)$ and $\bar{\vartheta}_y(t_k, T) = \frac{1}{N}\sum_i \vartheta_i^y\,(t_k, T)$ are recorded for every 10 MC steps so that the time interval of the data is $\Delta t\; = 10\tau = 10$ μs. The number of data points is $K = 10^6$ with $0 \le t_k \le (K-1)\Delta t$. $\bar{\vartheta}_x(t_k, T)$ and $\bar{\vartheta}_y(t_k, T)$ are statistically equivalent to each other.

Predicting physical quantities from the simulation

We calculated the one-sided power spectral density (PSD) $S_{\bar{\vartheta}_{x,y}}(\omega_j, T)$ and correlation function $C_{\bar{\vartheta}_{x,y}}(t_k, T)$ from $\bar{\vartheta}_{x,y}(t_k, T)$.

To increase the signal-to-noise ratio of PSD, we split the total time Γ into P segments $\bar{\vartheta}^p_{x,y}(t_k, T)$ of duration $\gamma = K_p\Delta t$ $\left(\Gamma = P\gamma, 0 \le p \le P-1, 0 \le t_k \le (K_p - 1)\Delta t\right)$. The PSD is calculated for each segment.

$$S_{\bar{\vartheta}^p_{x,y}}(\omega_j, T) = \frac{1}{\pi\gamma}\left|\Delta t \sum_{k=0}^{K_p-1} e^{-i\omega_j t_k}\bar{\vartheta}^p_{x,y}(t_k, T)\right|^2, \tag{M1}$$

where $\omega_j = \frac{2\pi}{\gamma} j$ $(0 \le j \le \frac{K_p}{2})$. PSD is obtained as the average of P segments.

$$S_{\bar{\vartheta}_{x,y}}(\omega_j, T) = \frac{1}{P}\sum_{p=0}^{P-1} S_{\bar{\vartheta}^p_{x,y}}(\omega_j, T)\,. \tag{M2}$$

Averages are further taken over 10 independent MC runs. We take as an error bar the standard error from independent runs. We used P values of $10, 10^2, 10^3$ to calculate PSDs of resolution $\Delta\omega/2\pi =\; 1, 10, 100$ Hz. The x-component $S_{\bar{\vartheta}_x}(\omega_j, T)$ for $T \le 0.15|J_1|$ and $T \le 0.30|J_1|$ are plotted in Figs. 2C and S1A, respectively. Fig. S2B shows the y-component $S_{\bar{\vartheta}_y}(\omega_j, T)$ equivalent to $S_{\bar{\vartheta}_x}(\omega_j, T)$.

The PSD $S_{\bar{\vartheta}_{x,y}}(\omega_j, T)$ is fitted by a function $A(T)\omega^{-\alpha(T)}$ in the frequency range $1\text{ Hz} \le \omega/2\pi \le 500$ Hz as shown in Figs. S1B and S2D. The obtained $\alpha(T)$ is plotted in Fig. 2E.

To address the low-frequency fluctuations, measured in the experiments, fluctuations above 1 kHz are filtered out from the time sequence $\bar{\vartheta}_{x,y}(t_k, T)$. In order to do this, Fourier components $\bar{\vartheta}_{x,y}(\omega_j, T) = \Delta t\sum_{k=0}^{K-1} e^{-i\omega_j t_k}\bar{\vartheta}_{x,y}(t_k, T)$ with $\omega_j = \frac{2\pi}{\Gamma} j\left(0 \le j \le \frac{K}{2}\right)$ are set to zero for $\omega_j/2\pi > 1$ kHz and brought back to time domain $\bar{\vartheta}'_{x,y}(t_k, T)$ by inverse Fourier transform. $\bar{\vartheta}'_{x,y}(t_k, T)$ is plotted in Figs. 2B and S2A.

The correlation function $C_{\bar{\vartheta}_{x,y}}(t_k, T)$ is calculated from $\bar{\vartheta}'_{x,y}(t_k, T)$ using the standard formula.

$$C_{\bar{\vartheta}_{x,y}}(t_k,T) = \frac{1}{l_{\mathrm{ave}}} \sum_{l=0}^{l_{\mathrm{ave}}-1} \bar{\vartheta}'_{x,y}(t_l,T)\,\bar{\vartheta}'_{x,y}(t_{l+k},T), \tag{M3}$$

where $l_{\mathrm{ave}} = 9 \times 10^5$. Averages are taken over 10 independent MC runs. The normalized correlation is calculated as $C_{\bar{\vartheta}_{x,y}}(t_k,T)/C_{\bar{\vartheta}_{x,y}}(0,T)$ and shown in Figs. 2D, S2C, and S10A.

The variance of the noise in Fig. 2F is calculated as

$$\sigma^2_{\bar{\vartheta}_{x,y}}(T) = \frac{1}{K}\sum_{k=0}^{K-1} \bar{\vartheta}'_{x,y}{}^{2}(t_k,T) - \left(\frac{1}{K}\sum_{k=0}^{K-1} \bar{\vartheta}'_{x,y}(t_k,T)\right)^2. \tag{M4}$$

Averages are taken over 10 independent MC runs.

Comparing the simulation to a realistic system

The correspondence between the MC time step and the actual time is decided as follows. In Fig. S3, we show the rate of a spin flip by an angle larger than 5 degrees at each temperature. The rate is calculated by counting the occurrence of such a spin flip in the first 5×10^4 MC steps of the time evolution of the spins in equilibrium. The rate of a spin flip larger than 5 degrees is 0.1 (MC step)$^{-1}$ around $T \sim 0.15|J_1|$. The elementary local relaxation process occurs at a timescale of the order $\tau_{\mathrm{ele}m} = 10$ (MC step) at low temperature. Ref. 5 reports AC susceptibility of $Ca_{10}Cr_7O_{28}$ in the form of Cole-Cole plot in the frequency range from 100 Hz to 20 kHz. Despite the deviation at low frequency, the Cole-Cole plot is on a semicircle. This suggests a relatively sharp distribution of relaxation time at high frequency, say $\omega/2\pi$ =10 kHz corresponding to ~ 10 μs. From this we estimate $\tau_{\mathrm{ele}m}$ to be at the order of 10 μs, namely 1 MC step = 1 μs.

Magnetization fluctuation is estimated from the average spin fluctuation. Consider N spins with magnitude s in a volume V. For large N, the fluctuation amplitude of average spin $\Delta\bar{\vartheta}$ and magnetization $\Delta B = \mu_0 \Delta M$ will be

$$\Delta\bar{\vartheta} \propto \frac{s\sqrt{N}}{N} = \frac{s}{\sqrt{N}}, \tag{M5}$$

$$\Delta B \propto \mu_0(2\mu_{\mathrm{B}})\frac{s\sqrt{N}}{V} = 2\mu_0\mu_{\mathrm{B}}\frac{N}{V}\Delta\bar{\vartheta}. \tag{M6}$$

In the simulation, we used $N^{\mathrm{sim}} = 1600$ spins of $s^{\mathrm{sim}} = 1$. This can be related to the experimental sample with N^{exp} spins of s^{exp} in volume V^{exp}.

$$\Delta\bar{\vartheta}^{\mathrm{exp}} = \frac{s^{\mathrm{exp}}}{s^{\mathrm{sim}}}\frac{\sqrt{N^{\mathrm{sim}}}}{\sqrt{N^{\mathrm{exp}}}}\Delta\bar{\vartheta}^{\mathrm{sim}}. \tag{M7}$$

$$\Delta B^{\mathrm{exp}} = 2\mu_0\mu_{\mathrm{B}}\frac{N^{\mathrm{exp}}}{V^{\mathrm{exp}}}\Delta\bar{\vartheta}^{\mathrm{exp}} = 2\mu_0\mu_{\mathrm{B}}\frac{s^{\mathrm{exp}}}{s^{\mathrm{sim}}}\frac{\sqrt{N^{\mathrm{sim}}N^{\mathrm{exp}}}}{V^{\mathrm{exp}}}\Delta\bar{\vartheta}^{\mathrm{sim}}. \tag{M8}$$

With $N^{\mathrm{sim}} = 1600, s^{\mathrm{sim}} = 1, N^{\mathrm{exp}} = 6.2 \times 10^{18}, s^{\mathrm{exp}} = 3/2,\ V^{\mathrm{exp}} = 2\ \mathrm{mm}^3$, the conversion factor to estimate B^{exp} from $\bar{\vartheta}^{\mathrm{sim}}$ is

$$2\mu_0\mu_\mathrm{B}\frac{S^{\mathrm{exp}}}{S^{\mathrm{sim}}}\frac{\sqrt{N^{\mathrm{sim}}N^{\mathrm{exp}}}}{V^{\mathrm{exp}}} = 1.7\times 10^{-9}\ \mathrm{T}. \qquad \text{(M9)}$$

The scale of temperature can also be compared. By setting $J_1 = -0.15$ meV and $J_2 = 0.042$ meV, $J_2/|J_1| = 0.28$ and J_2 is within the two error bars of the antiferromagnetic exchange energy 0.028 ± 0.008 meV in the empirical Hamiltonian of $Ca_{10}Cr_7O_{28}$ (6). This renders the variance peak temperature $T = 0.15|J_1| = 260$ mK, comparable to ~ 400 mK observed in the experiment.

Classical Monte Carlo simulations in $Ca_{10}Cr_7O_{28}$

As shown in Fig. 1A, the dominant intralayer ferromagnetic interaction in $Ca_{10}Cr_7O_{28}$ bundles up three spins on alternative plaquettes to form spin-3/2 state. Below the temperature scale set by the magnitude of these ferromagnetic interactions, a fairly accurate description of the low-energy properties can be obtained by working with effective S = 3/2 spins (7). S = 3/2 magnets can be described to a good approximation in classical terms so that (semi)classical simulations (7, 8) are effective. Indeed, classical Monte Carlo simulations and semiclassical simulations applied to $Ca_{10}Cr_7O_{28}$ (7, 8) have very successfully reproduced the liquid-like structure factors observed in experiments.

For $Ca_{10}Cr_7O_{28}$, we turned to the simplest generic model of spiral spin liquid as has been established by ref. 3. The simulation and the real $Ca_{10}Cr_7O_{28}$ both share the continuous contour of a spiral wave vector with an approximate $U(1)$ symmetry (5, 6, 9), which is the essence of the spiral spin liquid physics that renders two phases essentially the same (3), leading to the inhomogeneous spin texture of momentum vortices. This observation, together with the impressive wide-ranging agreement between the predicted SSL noise phenomenology in Fig. 2 and the measured $Ca_{10}Cr_7O_{28}$ noise data in Fig. 4, strongly indicates that our simulation captures the essence of spiral spin liquid dynamics in $Ca_{10}Cr_7O_{28}$.

In our simulation, spins are evolved via MC updates, and the dynamics due to an equation of motion is not considered. This is because the Hamiltonian (Eq. 1) does not give rise to a z-direction exchange field that will cause precession of the XY spins. Even if a z-direction field existed, it would only generate a very fast periodic precession of spins at 0.1 meV $\sim$ 10 ps that will be averaged out at the timescale of our MC simulation. We finally note that spiral spin liquid phase is found in both 2D XY spins on a square lattice (3) and 3D Heisenberg model on a honeycomb lattice (10), irrespective of the spin dimension and the underlying lattice symmetry.

Simulating spiral spin liquid model for Heisenberg spins

A Heisenberg Hamiltonian was initially proposed for $Ca_{10}Cr_7O_{28}$, although the possible existence of local anisotropic interactions (8, 11) is discussed in more recent work. To explore the effect of spin dimension, we also simulated the spiral spin liquid model for Heisenberg spins. The system size $L = 40$, Hamiltonian Eq. 1, and simulation procedure are completely identical as the XY-spin case, except that the spin configuration space is changed

from XY to a Heisenberg model. One independent MC run is performed, and the error bars of power spectral density and variance are calculated from the standard error of separated segments in Eqs. M2 and M4.

Also in the Heisenberg system, spins get more spatially correlated at low temperatures, as seen in the representative thermalized spin configurations at four different temperatures in Fig. S4A. The power spectral density $S_{\bar{\vartheta}_x}(\omega_j, T)$ in Fig. S4B exhibits an intense low-frequency fluctuation with a power-law frequency dependence. The noise variance $\sigma^2_{\bar{\vartheta}_{x,y,z}}(T)$ in Fig. S4C drops below a peak at $T \sim 0.1\,|J_1|$, with a relatively gentle slope of approximately T^1. These observations indicate that the qualitative features of spiral spin liquid noise described for the XY model in the main text occur irrespective of the spin dimension. However, momentum vortices are harder to identify in the Heisenberg case, and the spin noise predictions are quantitatively distinct. With the specific parameter set of our present simulation, the XY-spin result reproduces the experimental $Ca_{10}Cr_7O_{28}$ noise quantitatively significantly better. Optimization of simulation parameters which might alter its correspondence with the experiment, especially for the Heisenberg simulation, is left for future work.

Next developments in the simulation and theory

At $T > T^*$, the simulated power spectral density (Figs. S1A and S2B) starts showing flat white noise, while the experimental power spectral density (Fig. S7A) retains the power law behaviour. This indicates that real $Ca_{10}Cr_7O_{28}$ has additional factors that are not fully accounted for in the simulation, such as a larger system size or additional interactions. One possible candidate could be the existence of local anisotropic interactions (8, 11). This motivates further optimization of the spiral spin liquid Hamiltonian and simulation, and most importantly, finding an analytical theory of the spin noise spectrum of dynamical spin spirals.

Design of Noise Spectrometer/AC Susceptometer

A $^3He/^4He$ dilution refrigerator (Proteox MX) and a cryogen−free ^3He refrigerator (DRY ICE 300mK TERTIA) were used to carry out our experiments.

The spectrometer on the dilution refrigerator consists of a superconducting pickup coil that is enclosed in a superconducting excitation coil and connected to the SQUID (SP550). The pickup coil is wound on a macor sample holder with an inner diameter 1.6 mm and length 10 mm. A single NbTi wire forms two in-series counter-wound 10-turn pickup coils with a total inductance $L_\mathrm{p} = 0.75$ μH so that the external uniform flux is cancelled out. The SQUID input coil has an inductance of $L_\mathrm{i} = 1.74$ μH and a mutual inductance to the SQUID of $1/\mathcal{M}_\mathrm{i} = 0.19$ μA/Φ_0, as reported by the manufacturer (Quantum Design). The excitation coil is 10 mm long and has 101 turns of a NbTi wire. The whole circuitry is contained within two Nb cylinders covered by a mu-metal cylinder for magnetic flux shielding and is mounted on

the mK-plate of the refrigerator. The first Nb cylinder is provided by the SQUID manufacturer (Quantum Design), the second Nb shield has an inner diameter of 48 mm and 2 mm in thickness, and the mu-metal cylinder has an inner diameter of 58 mm and 1 mm in thickness. The whole dilution refrigerator is built on a 6-ton table that is mechanically isolated from external vibration. To accelerate the thermalization of the sample, a 0.1 mm diameter silver wire is attached to the sample by GE varnish and the other end is thermalized to the SQUID holder. The temperature of the sample and spectrometer is measured by a Rox thermometer which is mounted on the plate close to the SQUID assembly.

The spectrometer on the ^{3}He refrigerator is a superconducting pickup coil connected to the SQUID (SQ1200). 10 turns of NbTi wire with inductance $L_\mathrm{p} = 0.25$ μH is wound directly around a mm-scale, bar-shaped sample and fixed with GE varnish. This pickup coil circuitry is mounted on the SQUID chip with GE varnish. The SQUID input coil has an inductance of $L_\mathrm{i} = 1.3$ μH and a mutual inductance to SQUID of $1/\mathcal{M}_\mathrm{i} = 0.13$ μA$/\Phi_0$. For magnetic flux shielding, the pickup coil and SQUID are all contained within a Nb cylinder covered by a mu-metal cylinder. The spectrometer is mounted on a mechanical vibration isolator, hung under the bottom plate of the refrigerator. To accelerate thermalization of the sample, four 0.2 mm diameter brass wires are attached to the pickup circuitry by GE varnish and their other ends are in contact with a copper wire that exits the shielded region. The temperature of the sample and spectrometer is measured by a CX-1030 Cernox thermometer mounted on a vibration isolator, close to the SQUID circuitry.

Spectrometer calibration

The magnetic flux picked up from the sample $\Phi(t)$ and the output voltage of the SQUID $V_\mathrm{S}(t)$ are related by a simple constant

$$\Phi(t) = \frac{L_\mathrm{p} + L_\mathrm{i}}{\mathcal{M}_\mathrm{i}} \frac{1}{g} V_\mathrm{S}(t). \tag{M10}$$

$g = g_\mathrm{SQUID} g_\mathrm{preamp}$ consists of the conversion factor from flux to voltage in the SQUID g_SQUID and subsequent gain from the preamplifier g_preamp. g_SQUID is determined from the voltage jump due to a Φ_0-flux jump of the SQUID. For the highest sensitivity setting, $g_\mathrm{SQUID} = 0.73$ V$/\Phi_0$ for the SP550 SQUID and $g_\mathrm{SQUID} = 9.85$ V$/\Phi_0$ for the SQ1200 SQUID. Using these values, $V_\mathrm{S}(t)$ is converted to $\Phi(t)$.

$\Phi(t)$ is further converted to the magnetization $\mu_0 M(t)$ with the relation

$$\mu_0 M(t) = \frac{1}{NA} \Phi(t), \tag{M11}$$

where N is the number of turns of the pickup coil and A is the area of sample cross section. $N = 10$ for all setups. $A = 1$ mm^2 for Sample 1 in the dilution refrigerator.

$Ca_{10}Cr_7O_{28}$ Sample Preparation

As described in ref. 12, $Ca_{10}Cr_7O_{28}$ crystals are synthesized in a two-step process, including a solid-state reaction of $Ca_{10}Cr_7O_{28}$ powder and a travelling-solvent-floating-zone method for the single crystal growth. First, powder of $CaCO_3$ and Cr_2O_3 was mixed with a molar ratio of 3:1, sintered at 1000 °C for 24 hours, and rapidly quenched to room temperature. The sintering process was repeated after grinding and the addition of Cr_2O_3 powder until phase pure powder of $Ca_{10}Cr_7O_{28}$ was obtained. This powder was packed in a rod that is sintered at 1020 °C for 12 hours followed by a rapid quench to room temperature. This rod was used as a feed rod of the floating-zone growth, while a solvent was separately prepared following the same procedure from the powder of $CaCO_3$ and Cr_2O_3 with a molar ratio of 5:2. The growth was carried out in a 0.22 MPa oxygen pressure at 1 mm/hr using an optical floating zone furnace. The resulting single crystal was washed with HCl and then with H_2O. X-ray diffraction on a ground small piece confirms the phase purity. Fig. S5B shows the DC susceptibility of a typical single crystal measured in MPMS (Quantum Design). Fitting by $\chi = \chi_0 + \frac{C_{\mathrm{Curie}}}{T - T_{\mathrm{CW}}}$ in the temperature range $50\ \mathrm{K} \leq T \leq 250\ \mathrm{K}$ yields $T_{\mathrm{CW}} = +2.6$ K and an effective magnetic moment $\mu_{\mathrm{eff}} \approx 1.69\mu_{\mathrm{B}}$ that are comparable to the existing literature (6).

Photos of the three $Ca_{10}Cr_7O_{28}$ samples are shown in Fig. S5A. The long direction of the bar is identified to be the c-axis. Sample 1’ and Sample 2 are from the same growth while Sample 3 is from a separate growth. Sample 1 is obtained by later polishing down Sample 1’ to fit into the spectrometer of our dilution refrigerator. The measured magnetic noise from these three samples are consistent with each other, as described in the section ‘Repeatability of spin noise spectrum in different $Ca_{10}Cr_7O_{28}$ samples’.

Noise measurement

The results in the main text were measured in both the dilution refrigerator and the ^{3}He refrigerator. In the dilution refrigerator, the temperature of $Ca_{10}Cr_7O_{28}$ Sample 1 and the spectrometer was controlled by heaters from 100 mK to 500 mK in steps of 50 mK with the temperature stability of 1 mK. In the ^{3}He refrigerator, the temperature of the Sample 2 and the spectrometer was controlled by heaters from 300 mK to 800 mK in steps of 100 mK with the stability of 1 mK. In both setups, samples were thermalized for at least 15 minutes after the thermometer reading got stabilized at the target temperature. The overall circuit diagram for the noise measurement is shown in Fig. S6A. The output voltage of the SQUID $V_{\mathrm{S}}(t)$ was recorded by an effective 16-bit ADC (Moku:Pro) for 1000 s at a sampling rate of 20 kSa (a time interval of 50 μs). Between the SQUID and the ADC, a preamplifier (SR560) was used to amplify the SQUID output signal by an appropriate gain and to apply a 0.03 Hz 6 dB/Oct high-pass and 30 kHz 6 dB/Oct low-pass filter. For the SQUID output of the ^{3}He refrigerator setup, there was further filtering by a 5 kHz, 4-pole low-pass filter. With an identical setup, the SQUID background noise was measured for a nonmagnetic nylon sample at 800 mK.

To confirm the reproducibility, the noise signal of Sample 1', Sample 2, and Sample 3 was measured in the ^{3}He refrigerator. At temperatures from 275 mK to 800 mK in steps of 25 mK, the output voltage was recorded for 100 s at 1 MSa (a time interval of 1 μs) and 1.6 Hz AC coupling filter at the ADC input was used. A nonmagnetic nylon block of a comparable size was measured in the same condition in temperature steps of 100 mK.

An extended-bandwidth measurement was performed for Sample 2 in the ^{3}He refrigerator. The SQUID sensitivity was changed to medium $g_{\mathrm{SQUID}} = 0.985\ \mathrm{V}/\Phi_0$ and the frequency cutoff of the SQUID was extended to $f_{3\mathrm{dB}} \sim 300$ kHz by changing the internal capacitor of the SQUID feedback loop. At temperatures from 300 mK to 800 mK in steps of 100 mK, the output voltage of SQUID $V_{\mathrm{S}}(t)$ was recorded for 100 s at 1 MSa. The SQUID background noise was measured for a nonmagnetic nylon sample at 275 mK for 10 s at 1 MSa.

Noise analysis

In the main text, one-sided power spectral density (PSD) $S_\Phi(\omega_j, T)$ and correlation function $C_\Phi(t_k, T)$ are calculated from the experimental noise data $\Phi(t_k, T)$.

To increase the signal-to-noise ratio of the PSD, $\Phi(t_k, T)$ with a total time Γ =1000 s and a time interval $\Delta t = 50$ μs is split into P segments $\Phi^p(t_k, T)$ of duration $\gamma = K_p \Delta t$ $\left(\Gamma = P\gamma, 0 \le p \le P-1, 0 \le t_k \le (K_p - 1)\Delta t\right)$. The PSD for each segment is calculated from

$$S_{\Phi^p}(\omega_j, T) = \frac{1}{\pi\gamma}\left|\Delta t \sum_{k=0}^{K_p-1} e^{-i\omega_j t_k} \Phi^p(t_k, T)\right|^2, \tag{M12}$$

where $\omega_j = \frac{2\pi}{\gamma} j$ $(0 \le j \le \frac{K_p}{2})$. In a next step, the PSD is obtained as the average of P segments.

$$S_\Phi(\omega_j, T) = \frac{1}{P}\sum_{p=0}^{P-1} S_{\Phi^p}(\omega_j, T), \tag{M13}$$

with the standard error used as an error bar. $P = 10^2, 10^3, 10^4, 10^5$ was used to calculate the PSD of resolution $\Delta\omega/2\pi = 10^{-1}, 10^0, 10^1, 10^2$ Hz. The resulting PSD is plotted in Fig. 4A for 100 mK $\le T \le$ 400 mK and in Fig. S7A for 100 mK $\le T \le$ 800 mK.

In Fig. S7B, the PSD $S_\Phi(\omega_j, T)$ is fitted by a function $A(T)\omega^{-\alpha(T)}$ in the frequency range 0.1 Hz $\le \omega/2\pi \le$ 20 Hz. The obtained $\alpha(T)$ is plotted in Fig. 4D.

To get rid of the partial contribution of the electronic noise at high frequency and the slow temperature fluctuation at low frequency, the fluctuation below 0.05 Hz and that above 1 kHz are filtered out from the time sequence $\Phi(t_k, T)$. The filtering was done by setting the Fourier components $\Phi(\omega_j, T) = \Delta t \sum_{k=0}^{K-1} e^{-i\omega_j t_k} \Phi(t_k, T)$ below 0.05 Hz and above 1 kHz to zero, and then transforming the remaining signal $\Phi(\omega_j, T)$ back into the time domain $\Phi'(t_k, T)$ using an inverse Fourier transform. The filtered data is shown in Fig. 3B.

The correlation function $C_\Phi(t_k, T)$ is calculated from $\Phi'(t_k, T)$ using the standard formula

$$C_\Phi(t_k, T) = \frac{1}{l_{\rm ave}} \sum_{l=0}^{l_{\rm ave}-1} \Phi'(t_l, T)\, \Phi'(t_{l+k}, T)\,, \qquad \text{(M14)}$$

where $l_{\rm ave} = 1.9 \times 10^7$. The normalized correlation is calculated as $C_\Phi(t_k, T)/C_\Phi(0, T)$ and shown in Fig. 4B and Fig. S10B.

The variance of the noise, shown in Fig. 4E, is calculated as

$$\sigma_\Phi^2(T) = \frac{1}{K}\sum_{k=0}^{K-1} \Phi'^2(t_k, T) - \left(\frac{1}{K}\sum_{k=0}^{K-1} \Phi'(t_k, T)\right)^2 . \qquad \text{(M15)}$$

The fitting by $\sigma_\Phi^2(T) \propto T^\beta$ is performed in the temperature range of 100 mK $\leq T \leq$ 300 mK to give $\beta = 2.3 \pm 0.1$.

The noise data of Sample 1', Sample 2, and Sample 3, measured with a total time Γ =100 s and a time interval $\Delta t = 1$ μs, is used to calculate the PSD with $P = 10^3$ that is shown in Fig. S5C. The variance is calculated after filtering the fluctuation below 0.05 Hz and above 10 kHz, as shown in Fig. S5D.

In Fig. S8, we show the PSD of Sample 2 and Nylon in the full frequency range from 0.1 Hz to 50 kHz. The frequency range $\omega/2\pi$ below 3 kHz is calculated from the noise data with Γ =1000 s and $\Delta t = 50$ μs using $P = 10^2, 10^3, 10^4, 10^5$. The frequency range above 3 kHz is calculated from the extended-frequency measurement data of Sample 2 (Nylon) with $\Gamma = 100$ s (10 s) and $\Delta t = 1$ μs using $P = 10^5, 10^6$ ($10^4, 10^5$). The noise of Nylon corresponding to the background noise level is enhanced by ~10 times for the extended-frequency measurement. In Fig. S8, the PSD of Sample 2 above 3 kHz is plotted after subtraction of the Nylon noise. The PSD of Sample 2 below and above 3 kHz smoothly connects to each other.

Noise data from different spectrometers

The experimental noise data $\Phi(t, T)$ in Fig. 3B was measured in two different samples Sample 1 and Sample 2 for different temperature ranges. The amplitude of magnetization noise $B(t)$ is dependent on a measured sample volume, and the amplitude of $\Phi(t)$ is further dependent on the area of the sample cross section. Thus, a scale factor is naturally required to patch up the results for two samples with different geometry. In principle, one can only match the scale of either $\Phi(t)$ or $B(t)$. Throughout the paper, the matching of $\Phi(t)$ is prioritized, and $B(t)$ is converted from $\Phi(t)$ using the geometry of Sample 1 for both samples.

Fig. S9 shows the comparison of power spectral density from the two samples at an overlapping temperature 300 mK. Here, the $\Phi(t)$ of Sample 2 is scaled from an original value by a factor of 0.95 (i.e. $S_\Phi(\omega)$ by 0.95^2) so that two data coincide.

Repeatability of spin noise spectrum in different $Ca_{10}Cr_7O_{28}$ samples

The noise measurement was repeated in ^{3}He refrigerator for three $Ca_{10}Cr_7O_{28}$ samples and a nonmagnetic nylon block of a comparable size shown in Fig. S5A. In Fig. S5C, we show the power spectral density of each sample at temperatures of 300 mK, 500 mK, 700 mK, and 800 mK. All $Ca_{10}Cr_7O_{28}$ samples have a scale-invariant power spectral density. The nylon noise floor barely changes over temperature and remains much smaller than the signal of $Ca_{10}Cr_7O_{28}$. Fig. S5D shows the temperature dependence of variance. All $Ca_{10}Cr_7O_{28}$ samples show a peak around $T \sim 400$ mK. Thus, the reported behavior of $Ca_{10}Cr_7O_{28}$ noise is robust.

AC susceptibility measurement

We performed the AC susceptibility measurement of Sample 1 in the dilution refrigerator for temperatures from 100 mK to 500 mK in 50 mK steps with the stability of 1 mK. At each target temperature, the sample was thermalized for 20 minutes after the thermometer reading got stabilized. The overall circuit diagram for the AC susceptibility measurement is shown in Fig. S6B. An AC magnetic field with a root-mean-square magnitude of $B_{\mathrm{exc}} = 60$ nT in the frequency range 0.1 Hz $\leq \omega/2\pi \leq$ 101 Hz was applied using the reference output of the lock-in amplifier (SR830) in series of a 20 kΩ resistor and the excitation coil circuitry. The output voltage of the SQUID at the lowest sensitivity $g_{\mathrm{SQUID}} = 7.3\ \mathrm{mV}/\Phi_0$ was fed into the lock-in amplifier to measure the in-phase and out-of-phase components. At each frequency, 10 measurements were performed and the results were averaged. The zero phase was set by performing a calibration experiment with a superconducting indium wire of the size comparable to the sample. The time constant of the lock-in amplifier was set at 30 s, 10 s, 3 s, 300 ms for frequencies 0.1-0.3 Hz, 0.5-0.9 Hz, 1-11 Hz, and 21-101 Hz, respectively. The low-pass filter was set at 18 dB/oct for all frequencies. The sensitivity of the lock-in amplifier was set to an appropriate value between 10 mV and 50 mV for different temperatures.

AC susceptibility analysis

The out-of-phase output of the lock-in amplifier $V_Y(\omega)$ was converted to magnetization $M_Y(\omega)$ using Eq. 3 and then to the imaginary susceptibility by

$$\chi''(\omega) = -\frac{\mu_0 M_Y(\omega)}{B_{\mathrm{exc}}}. \tag{M16}$$

The fluctuation-dissipation theorem relates the imaginary part of the susceptibility $\chi''(\omega, T)$ with the one-sided power spectral density of magnetization noise $S_M(\omega, T)$ as

$$\chi''(\omega, T) = \mu_0 V \frac{\pi\omega S_M(\omega, T)}{2k_{\mathrm{B}}T}, \tag{M17}$$

where T is the temperature and V is the measured volume of the sample that is set to 2 mm^3 here (13). In Fig. 3C, the right-hand side is plotted against the left-hand side. 11-101 Hz data of $\chi''(\omega, T)$ is matched to 10-100 Hz data of $S_M(\omega, T)$ with $\Delta\omega/2\pi = 10$ Hz.

Spin noise and AC susceptibility

Spin noise power spectral density $S_M(\omega, T)$ and imaginary part of AC magnetic susceptibility $\chi''(\omega, T)$ are related by the fluctuation-dissipation theorem (FDT) in an equilibrium state

$$S_M(\omega, T) = \frac{1}{V\mu_0}\frac{2k_\mathrm{B}T}{\pi\omega}\chi''(\omega, T). \tag{M18}$$

When FDT is valid, both measurements should yield the same quantities, although there is a key difference that spin noise measurement does not require the application of any magnetic field. This is beneficial in studies of spin liquids where a small field could alter an underlying spin state, and also a simpler design of the spectrometer makes it a promising approach to performing local measurements and extending the measurement frequency.

When the relaxational time far exceeds the experimental time, for example in a spin glass state, the FDT is expected to break down (13). We recently exemplified this situation in $Dy_2Ti_2O_7$ (14). Such FDT breaking is not observed in $Ca_{10}Cr_7O_{28}$ at least down to 100 mK and 0.1 Hz.

Literature searches do not find reports of spin noise measurement (i.e. magnetization noise measurement) in well-known spin glasses CuMn or AuFe, although they do exist in some spin glass compounds such as $(Al_2O_3)_{0.1}(MnO)_{0.5}$ $(SiO_2)_{0.4}$ (15) and $Eu_{0.4}Sr_{0.6}S$ (16). These papers support the validity of FDT, but only based on a few high-frequency points well above 1 Hz. A comprehensive study of FDT in these conventional spin glasses is motivated.

Spin noise and charge noise

Spin noise spectroscopy can be compared to the charge noise spectroscopy. SQUID spin noise spectroscopy measures the fluctuation of magnetization $M(t)$, and is controlled by the spin precession and relaxation. On the other hand, charge noise spectroscopy measures the fluctuation of current noise density $J(t)$, which corresponds to the oscillation and scattering of charged particles momentum.

By defining an electric susceptibility $P = \chi_e D$, where P is the polarization and D is the electric displacement field, the polarization noise power spectral density is expressed as

$$S_P(\omega, T) = \frac{\varepsilon_0}{V}\frac{2k_\mathrm{B}T}{\pi\omega}\chi_e''(\omega, T), \tag{M19}$$

where ε_0 is vacuum permittivity. This is an electric version of Eq. M18.

In the similar manner, the noise of current density J can be expressed by an AC conductivity. By defining the AC conductivity as $J = (\sigma/\varepsilon_0)D$, the current density power spectral density is

$$S_J(\omega, T) = \frac{1}{V}\frac{2k_\mathrm{B}T}{\pi}\sigma'(\omega, T). \tag{M20}$$

Eqs. M19 and M20 would be most relevant for dielectric material. We note that SQUID spin noise spectrometer is also sensitive to charge noise from a metallic compound, but in a more

complex fashion because here the current generates magnetic field through the Biot-Savert law.

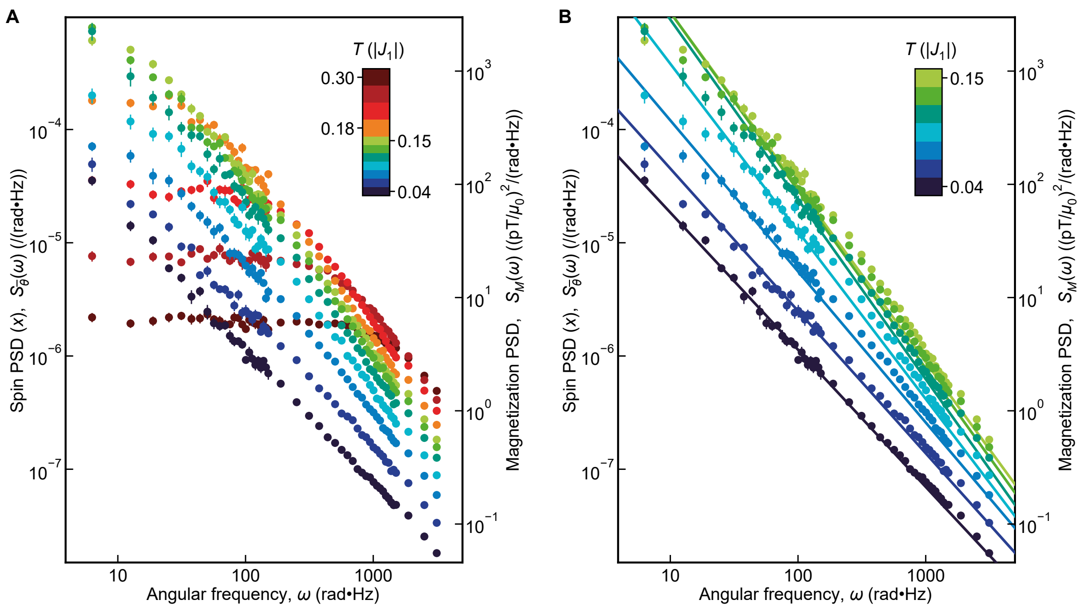

Fig. S1. The simulated spin noise power spectral density $S_{\bar{\vartheta}_x}(\omega, T)$ fit by $A(T)\omega^{-\alpha(T)}$.

A. The power spectral density of simulated spiral spin liquid noise $S_{\bar{\vartheta}_x}(\omega, T)$ for eleven selected temperatures in the range of $0.04|J_1| \leq T \leq 0.30|J_1|$. 1 MC time step is set at $\tau = 1\ \mu s$. Error bars are the standard error of the independent MC simulation runs. The anticipated power spectral density of magnetization noise $S_M(\omega, T)$ is shown on the right-hand axis as estimated from calculations described in this document.

B. Fitting of the simulated power spectral density $S_{\bar{\vartheta}_x}(\omega, T) = A(T)\omega^{-\alpha(T)}$ in the range of $1\ \text{Hz} \leq \omega/2\pi \leq 500\ \text{Hz}$ for seven selected temperatures in the range of $0.04|J_1| \leq T \leq 0.15|J_1|$.

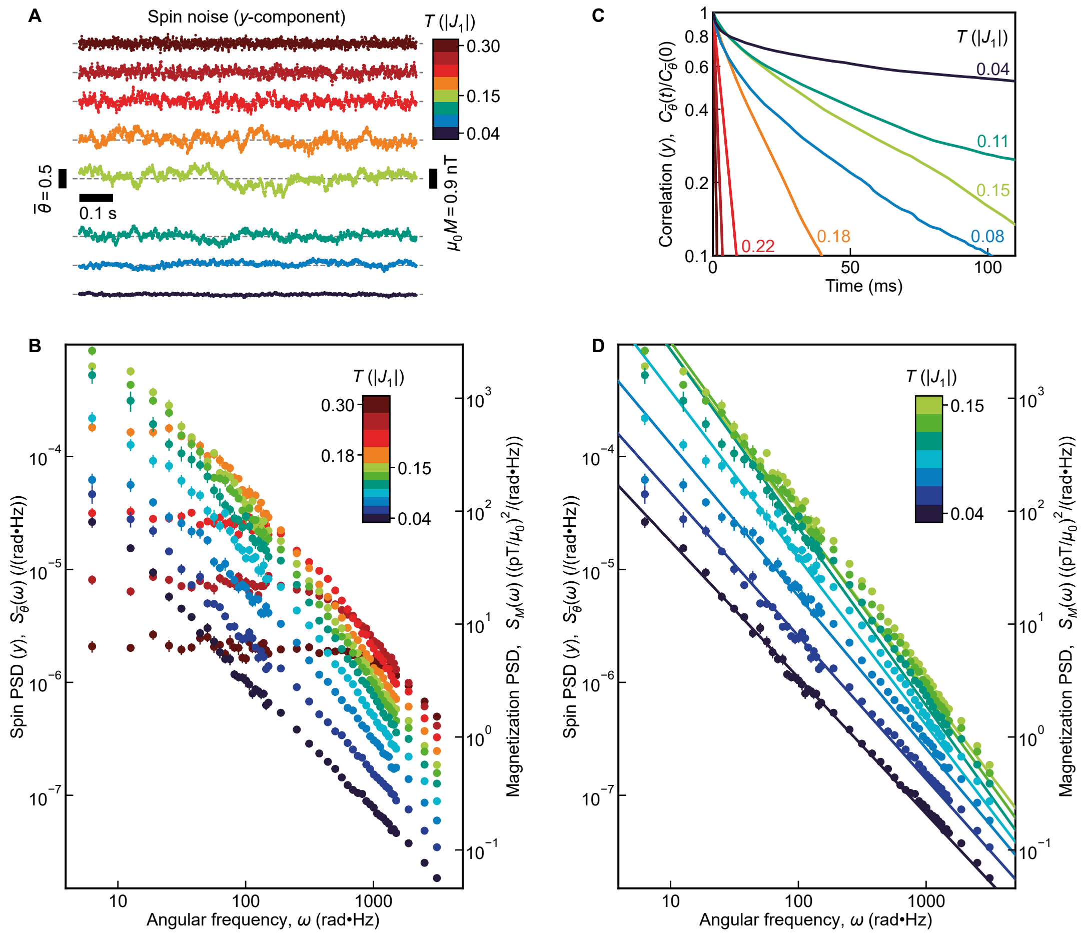

Fig. S2. y-component of average spin from MC simulations of spiral spin liquid noise.

A. MC-predicted time sequence of average y-component spin $\bar{\vartheta}_y(t,T) = \frac{1}{N}\sum_{\boldsymbol{r}} \vartheta_y(\boldsymbol{r},t,T)$ at eight temperatures for $N = 40 \times 40$ sites, equivalent to $\bar{\vartheta}_x(t,T)$ in Fig. 2B. We take 1 MC time step to be $\tau = 1$ μs. $\bar{\vartheta}_y(t,T)$ is down sampled for visual clarity to every 500 MC steps so that time intervals shown are $500\tau = 500$ μs. The frequency component above 1 kHz is filtered out.

B. The power spectral density of simulated average y-component spin $S_{\bar{\vartheta}_y}(\omega,T)$ for the eleven selected temperatures, comparable to $S_{\bar{\vartheta}_x}(\omega,T)$ in Figs. 2C and S1A. 1 MC time step is $\tau = 1$ μs. Error bars are the standard error of the independent MC simulation runs. The anticipated power spectral density of magnetization noise $S_M(\omega,T)$ is shown on the right-hand axis as estimated from calculations described in this document.

C. The correlation function of average y-component spin $C_{\bar{\vartheta}_y}(t,T)/C_{\bar{\vartheta}_y}(0,T)$ comparable to $C_{\bar{\vartheta}_x}(t,T)/C_{\bar{\vartheta}_x}(0,T)$ in Fig. 2D.

D. Fitting of the simulated power spectral density $S_{\bar{\vartheta}_y}(\omega,T) = A(T)\omega^{-\alpha(T)}$ in the range of $1\text{ Hz} \le \omega/2\pi \le 500\text{ Hz}$ for seven selected temperatures in the range of $0.04|J_1| \le T \le 0.15|J_1|$.

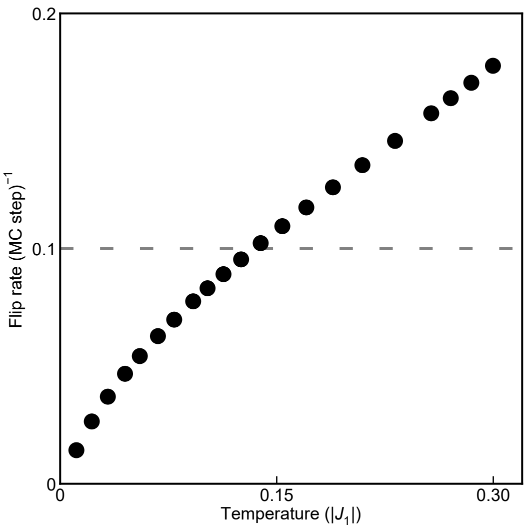

Fig. S3. The rate of a spin flip by an angle larger than 5 degrees in the Monte Carlo simulation.

The rate of a spin flip by an angle larger than 5 degrees at each temperature counted in the first 5×10^4 MC steps of the time evolution of $N = 40 \times 40$ spins in equilibrium. The rate is 0.1 (MC step)$^{-1}$ around $T \sim 0.15|J_1|$, suggesting an elementary local relaxation process timescale of the order $\tau_{\mathrm{ele}m} = 10$ (MC step).

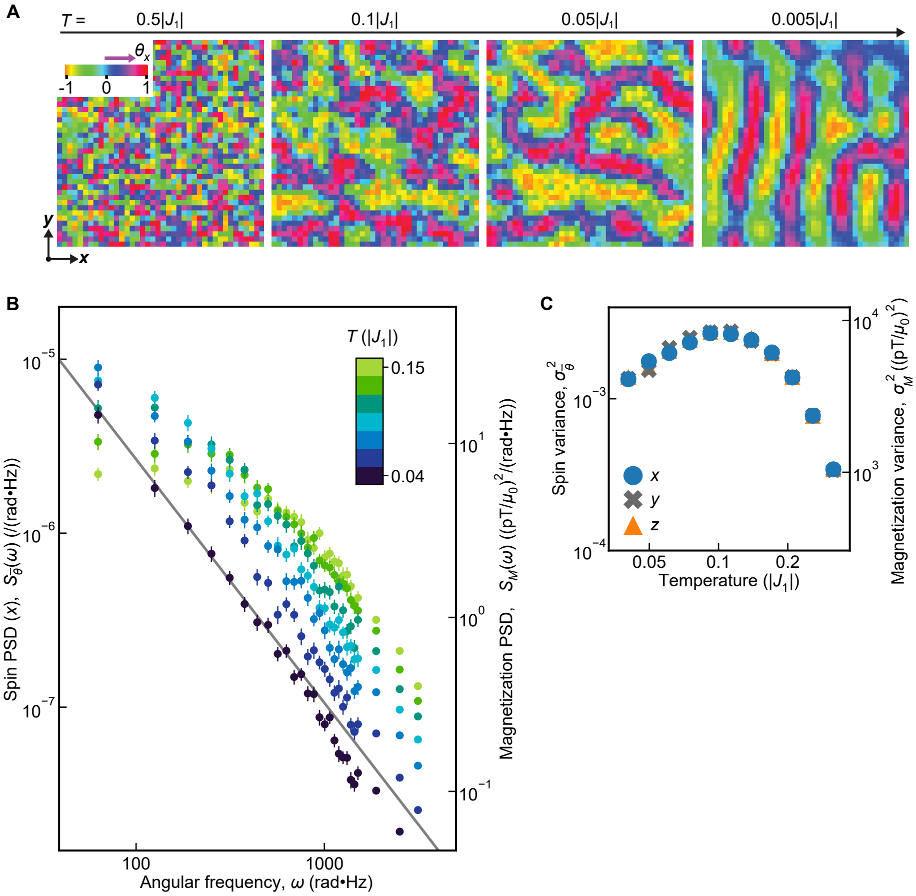

Fig. S4. MC simulations of spiral spin liquid noise for Heisenberg spins.
A. MC simulations of a snapshot of $\theta_x(\boldsymbol{r})$ on the SSL model of Eq. 1 using a square lattice with $N = 40 \times 40$ sites $\boldsymbol{r}$, each site with a three-dimensional spin unit vector $\boldsymbol{\vartheta}(\boldsymbol{r})$. Each snapshot is for a different temperature so that this sequence of SSL simulation snapshots is for approximately $T = 0.5|J_1|, 0.1|J_1|, 0.05|J_1|$ and $0.005|J_1|$.
B. From the time sequences of average x-component spin $\bar{\vartheta}_x(t,T)$ in the three-dimensional-spin simulation, the power spectral density of simulated noise $S_{\bar{\vartheta}_x}(\omega,T)$ is derived as a function of temperature T and shown for seven selected temperatures. Again we take 1 MC time step to be $\tau = 1$ μs. Here the error bars are the standard error of separated segments. The anticipated power spectral density of magnetization noise $S_M(\omega,T)$ is shown on the right-hand axis as estimated from calculations described in this document. The spectrum shows a powerful low-frequency noise down to $\omega/2\pi = 10$ Hz with a diminishing power below $T \sim 0.1|J_1|$. $\omega^{-1.4}$ line (gray) is drawn as a guide to the eye.
C. From the time sequences $\bar{\vartheta}_{x,y,z}(t,T)$ (the frequency component above 1 kHz is filtered out), the variance of simulated SSL noise $\sigma^2_{\bar{\vartheta}_{x,y,z}}(T)$ is presented. The variance peaks around $T = 0.1|J_1|$ and diminishes approximately as T^1.

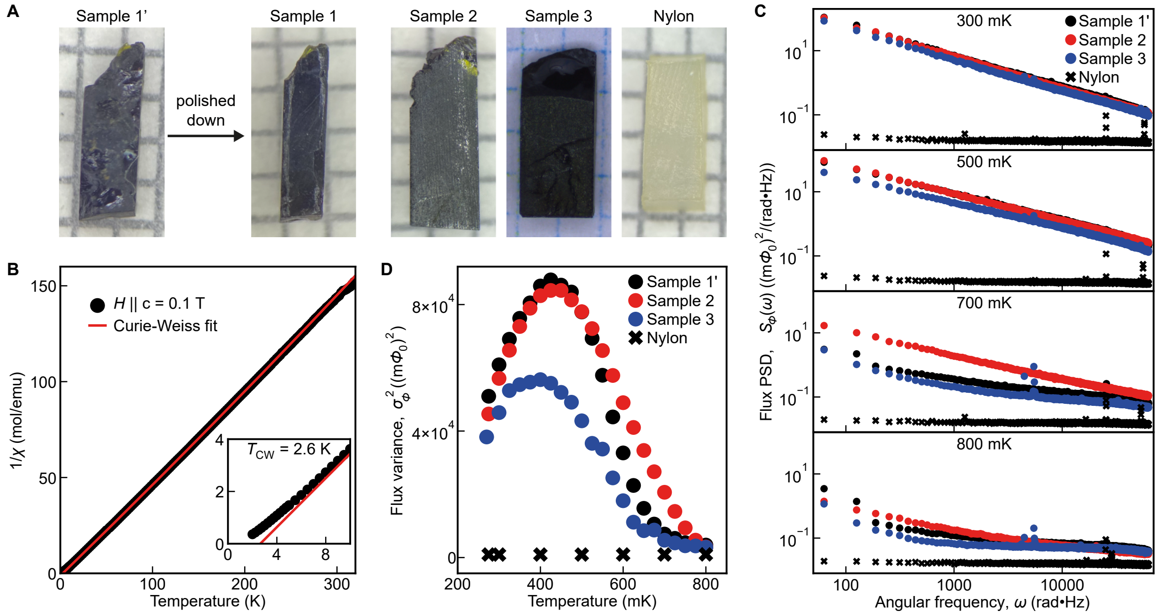

Fig. S5. Single crystals of $Ca_{10}Cr_7O_{28}$ and comparison of their noise signals.

A. Photos of the three $Ca_{10}Cr_7O_{28}$ samples and a nonmagnetic nylon block of a comparable size. Sample 1 is obtained by polishing down Sample 1'.

B. DC susceptibility of Sample 3 measured by MPMS. The Curie-Weiss fitting by $\chi = \chi_0 + \frac{C_{\text{Curie}}}{T - T_{\text{CW}}}$ (red line) yields $T_{\text{CW}} = +2.6$ K and $C_{\text{Curie}} = 2.1$ K•emu/mol corresponding to $\mu_{\text{eff}} = 1.69\mu_{\text{B}}$.

C. Comparison of the flux noise power spectral density $S_\Phi(\omega, T)$ of the three $Ca_{10}Cr_7O_{28}$ samples and the nylon block at four temperatures. All the $Ca_{10}Cr_7O_{28}$ samples generate a strong magnetic noise above the Nylon signal corresponding to a background noise.

D. Comparison of the flux noise variance $\sigma_\Phi^2(T)$ of the three $Ca_{10}Cr_7O_{28}$ samples and the nylon block. All the $Ca_{10}Cr_7O_{28}$ samples show a peak at $T \sim 400$ mK.

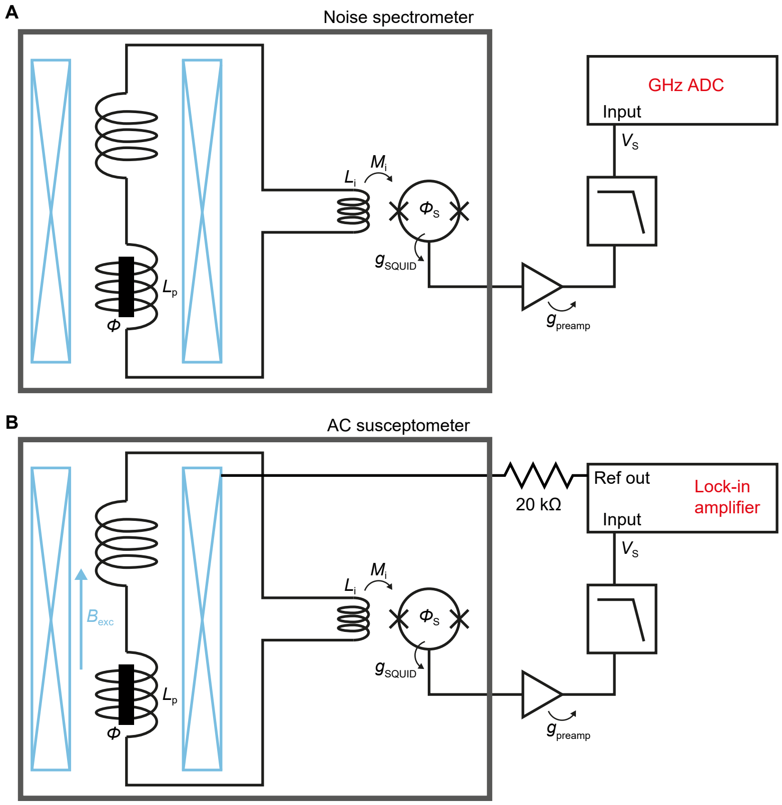

Fig. S6. Schematic circuit diagrams of the experiment.

A. Circuit diagram of the noise measurement.

B. Circuit diagram of the AC susceptibility measurement.

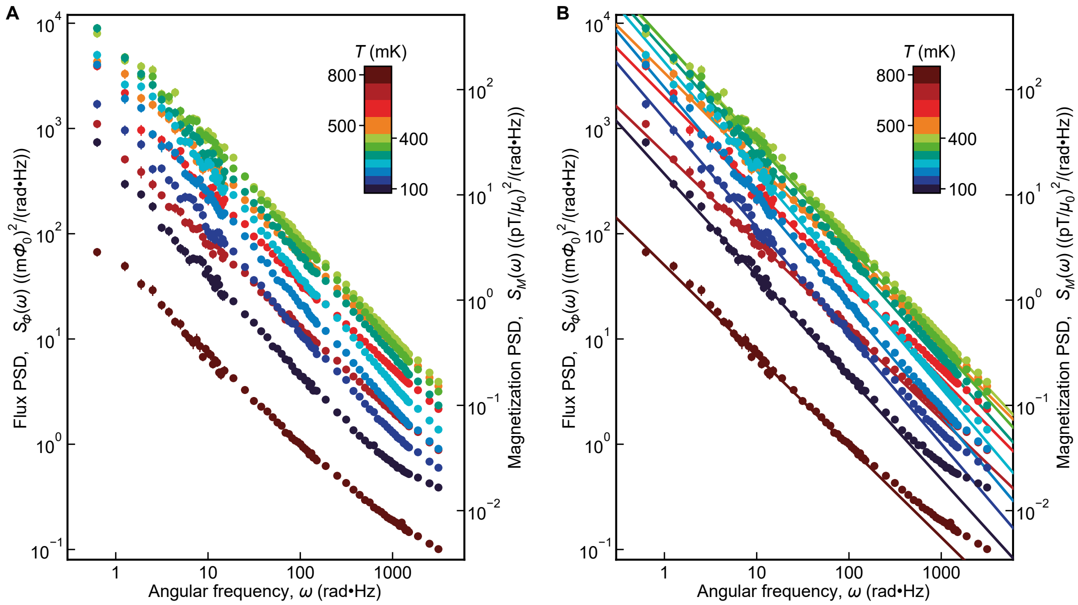

Fig. S7. Experimental spin noise power spectral density of $Ca_{10}Cr_7O_{28}$ $S_{\Phi}(\omega, T)$ and fitting by $A(T)\omega^{-\alpha(T)}$.

A. Experimental power spectral density of $Ca_{10}Cr_7O_{28}$ $S_{\Phi}(\omega, T)$ in the full measured-temperature range of 100 mK $\leq T \leq$ 800 mK. The equivalent power spectral density of magnetic field noise at the sample $S_M(\omega, T)$ is presented on right hand axis.

B. Fitting of experimental power spectral density $S_{\Phi}(\omega, T) = A(T)\omega^{-\alpha(T)}$ in the frequency range 0.1 Hz $\leq \omega/2\pi \leq$ 20 Hz for 100 mK $\leq T \leq$ 800 mK.

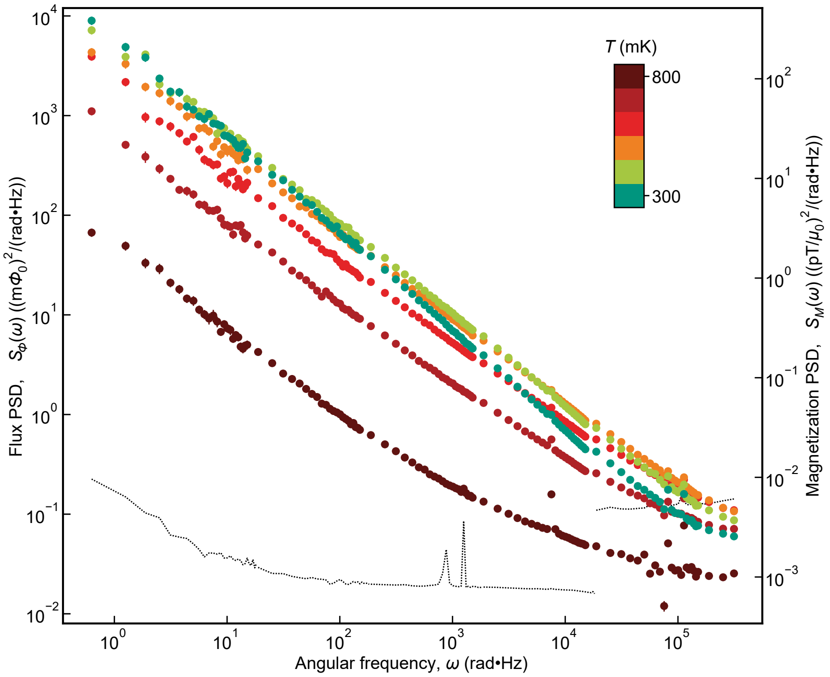

Fig. S8. Experimental spin noise power spectral density of $Ca_{10}Cr_7O_{28}$ in an extended frequency range.

Experimental power spectral density of $Ca_{10}Cr_7O_{28}$ in an extended frequency range of $0.1\ \text{Hz} \leq \omega/2\pi \leq 50\ \text{kHz}$ for six temperatures. The background noise measured for a nonmagnetic Nylon is plotted as a black-dotted line. The frequency range $\omega/2\pi$ above 3 kHz is from an extended-frequency measurement that enhances the noise floor. Above 3 kHz, $Ca_{10}Cr_7O_{28}$ noise is plotted after a subtraction by the background noise.

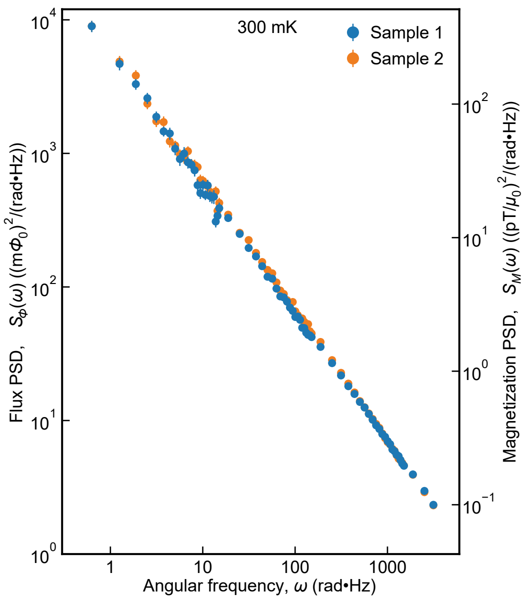

Fig. S9. Comparison of experimental power spectral density from Sample 1 and Sample 2.

The comparison of power spectral density $S_\Phi(\omega, T)$ from Sample 1 and Sample 2. $S_\Phi(\omega, T)$ of Sample 2 is scaled by 0.95^2 so that the value of two flux power spectral densities agrees. The equivalent spontaneous magnetization noise $S_M(\omega, T)$ on the right axis is converted using the geometry of Sample 1.

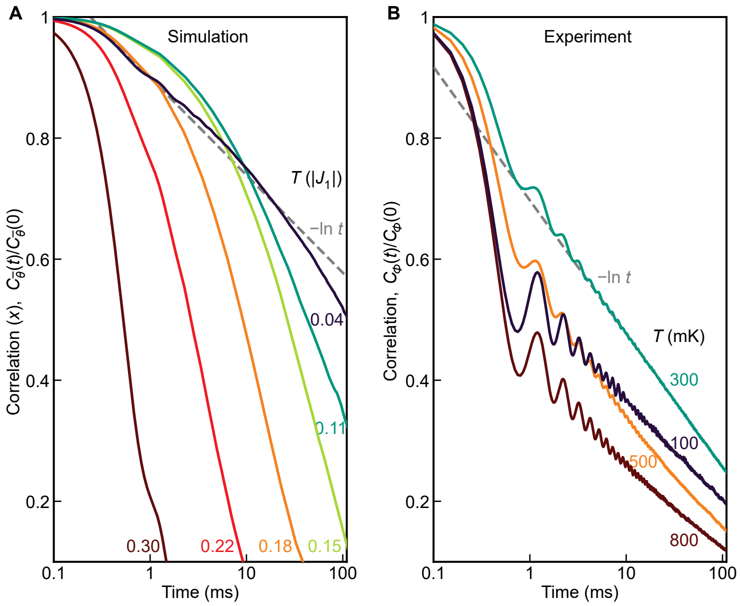

Fig. S10. The correlation functions in ln *t* scale.

A. The simulated correlation function $C_{\bar{\vartheta}_x}(t,T)/C_{\bar{\vartheta}_x}(0,T)$ of x-component spin noise (same quantity as Fig. 2D), plotted with a log scale horizontal axis. The correlation function gradually approaches to an almost linear behaviour $\propto -\ln t$ (gray dashed line) at low temperature.

B. The experimental correlation function $C_\Phi(t_k,T)/C_\Phi(0,T)$ of flux noise $\Phi(t)$ (same quantity as Fig. 4B), plotted with a log scale horizontal axis. The correlation function shows a linear behaviour$\propto -\ln t$ (gray dashed line).

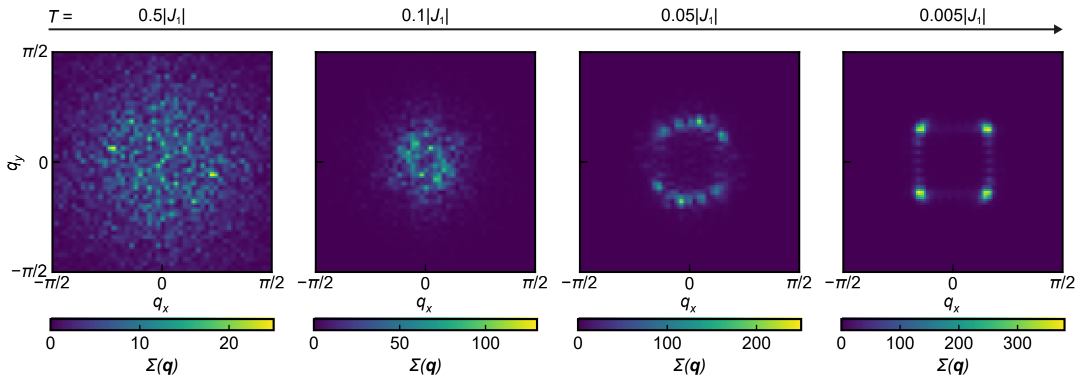

Fig. S11. Structure factor of spiral spin liquid Monte Carlo simulations

Structure factor $\Sigma(\boldsymbol{q})$ of the spiral spin liquid simulation, which is calculated from each spin pattern shown in Fig. 2A corresponding to $T = 0.5|J_1|$, $0.1|J_1|$, $0.05|J_1|$ and $0.005|J_1|$.

Movie S1 (separate file). Visualization of the spin equilibration process in an $L = 100$ system.
The evolution of spin configuration as the $L = 100$ system is equilibrated by a total of 6×10^6 MC steps. The color of the pixel represents the direction of the spin with the same color code as Fig. 2A. 10^4 MC steps are performed between each picture frame.

Movie S2 (separate file). Visualization of the spin equilibration process in an $L = 40$ system.
The evolution of spin configuration as the $L = 40$ system is equilibrated by a total of 6×10^6 MC steps. The color of the pixel represents the direction of the spin with the same color code as Fig. 2A. 10^4 MC steps are performed between each picture frame.

SI References

1 S. Chatterjee, J. F. Rodriguez-Nieva, E. Demler, Diagnosing phases of magnetic insulators via noise magnetometry with spin qubits. *Phys. Rev. B* **99**, 104425 (2019).

2 J. Y. Khoo, F. Pientka, P. A. Lee, I. S. Villadiego, Probing the quantum noise of the spinon Fermi surface with NV centers. *Phys. Rev. B* **106**, 115108 (2022).

3 H. Yan, J. Reuther, Low-energy structure of spiral spin liquids. *Phys. Rev. Res.* **4**, 023175 (2022).

4 L. W. Lee, A. P. Young, Large-scale Monte Carlo simulations of the isotropic three-dimensional Heisenberg spin glass. *Phys. Rev. B* **76**, 024405 (2007).

5 C. Balz *et al.*, Physical realization of a quantum spin liquid based on a complex frustration mechanism. *Nat. Phys.* **12**, 942–949 (2016).

6 C. Balz *et al.*, Magnetic Hamiltonian and phase diagram of the quantum spin liquid $Ca_{10}Cr_7O_{28}$. *Phys. Rev. B* **95**, 174414 (2017).

7 S. Biswas, K. Damle, Semiclassical theory for liquidlike behavior of the frustrated magnet $Ca_{10}Cr_7O_{28}$. *Phys. Rev. B* **97**, 115102 (2018).

8 R. Pohle, H. Yan, N. Shannon, Theory of $Ca_{10}Cr_7O_{28}$ as a bilayer breathing-kagome magnet: Classical thermodynamics and semiclassical dynamics. *Phys. Rev. B* **104**, 024426 (2021).

9 J. Sonnenschein *et al.*, Signatures for spinons in the quantum spin liquid candidate $Ca_{10}Cr_7O_{28}$. *Phys. Rev. B* **100**, 174428 (2019).

10 T. Shimokawa, H. Kawamura, Ripple State in the Frustrated Honeycomb-Lattice Antiferromagnet. *Phys. Rev. Lett.* **123**, 057202 (2019).

11 J. Crossley, C. Hooley, Origin of the intermediate-temperature magnetic specific heat capacity in the spin-liquid candidate $Ca_{10}Cr_7O_{28}$. *Phys. Rev. B* **109**, 165138 (2024).

12 C. Balz *et al.*, Crystal growth, structure and magnetic properties of $Ca_{10}Cr_7O_{28}$. *J. Phys.: Condens. Matter* **29**, 225802 (2017).

13 V. Raban, L. Berthier, P. C. W. Holdsworth, Violation of the fluctuation-dissipation theorem and effective temperatures in spin ice. *Phys. Rev. B* **105**, 134431 (2022).

14 J. Dasini *et al.*, Discovery of Dynamical Heterogeneity in a Supercooled Magnetic Monopole Fluid. arXiv [Preprint] (2024). https://arxiv.org/abs/2408.00460 (accessed 28 October 2024)
15 M. Ocio, H. Bouchiat, P. Monod, Observation of 1/*f* magnetic fluctuations in spin glasses. *J. Magn. Magn. Mater.* **54–57**, 11–16 (1986).
16 W. Reim, R. H. Koch, A. P. Malozemoff, M. B. Ketchen, H. Maletta, Magnetic Equilibrium Noise in Spin-Glasses: $Eu_{0.4}Sr_{0.6}S$. *Phys. Rev. Lett.* **57**, 905–908 (1986).
